# Genome-wide identification and functional analysis of Apobec-1-mediated C-to-U RNA editing in mouse small intestine and liver

**DOI:** 10.1186/gb-2014-15-6-r79

**Published:** 2014-06-19

**Authors:** Valerie Blanc, Eddie Park, Sabine Schaefer, Melanie Miller, Yiing Lin, Susan Kennedy, Anja M Billing, Hisham Ben Hamidane, Johannes Graumann, Ali Mortazavi, Joseph H Nadeau, Nicholas O Davidson

**Affiliations:** 1 Department of Medicine, Washington University St Louis, St Louis, MO 63110, USA; 2 Department of Developmental and Cell Biology and Center for Complex Biological Systems, University of California Irvine, Irvine, CA 92697, USA; 3 Pacific Northwest Research Institute, Seattle, WA 98122, USA; 4 Departments of Surgery, Washington University St Louis, St Louis, MO 63110, USA; 5 Proteomics Core, Weill Cornell Medical College in Qatar, Doha, Qatar

## Abstract

**Background:**

RNA editing encompasses a post-transcriptional process in which the genomically templated sequence is enzymatically altered and introduces a modified base into the edited transcript. Mammalian C-to-U RNA editing represents a distinct subtype of base modification, whose prototype is intestinal apolipoprotein B mRNA, mediated by the catalytic deaminase Apobec-1. However, the genome-wide identification, tissue-specificity and functional implications of Apobec-1-mediated C-to-U RNA editing remain incompletely explored.

**Results:**

Deep sequencing, data filtering and Sanger-sequence validation of intestinal and hepatic RNA from wild-type and *Apobec*-1-deficient mice revealed 56 novel editing sites in 54 intestinal mRNAs and 22 novel sites in 17 liver mRNAs, all within 3′ untranslated regions. Eleven of 17 liver RNAs shared editing sites with intestinal RNAs, while 6 sites are unique to liver. Changes in RNA editing lead to corresponding changes in intestinal mRNA and protein levels for 11 genes. Analysis of RNA editing *in vivo* following tissue-specific Apobec-1 adenoviral or transgenic Apobec-1 overexpression reveals that a subset of targets identified in wild-type mice are restored in *Apobec*-1-deficient mouse intestine and liver following Apobec-1 rescue. We find distinctive polysome profiles for several RNA editing targets and demonstrate novel exonic editing sites in nuclear preparations from intestine but not hepatic apolipoprotein B RNA. RNA editing is validated using cell-free extracts from wild-type but not *Apobec*-1-deficient mice, demonstrating that Apobec-1 is required.

**Conclusions:**

These studies define selective, tissue-specific targets of Apobec-1-dependent RNA editing and show the functional consequences of editing are both transcript- and tissue-specific.

## Background

There is considerable interest in understanding both the repertoire of and mechanisms for RNA-DNA differences reported from deep sequencing (RNA-seq) of mammalian transcriptomes [1-6]. Among the mechanisms for RNA-DNA differences is RNA editing, in which genomically templated RNA sequences are enzymatically altered. The most prevalent type of editing involves a base change from adenosine to inosine (A-to-I), mediated by adenosine deaminases acting on (double-stranded) RNA (ADARs) [7]. A second, much less prevalent type of RNA editing involves deamination of cytidine to uridine (C-to-U) in single-stranded RNA, mediated by Apobec-1, a member of the APOBEC family of cytidine deaminases [8]. The prototype for mammalian C-to-U RNA editing is apolipoprotein B (apoB) RNA, where Apobec-1-mediated deamination of a **C**AA codon introduces a translational termination (**U**AA) codon in the edited transcript. ApoB mRNA editing is a critical adaptive pathway for lipid transport in both the mouse intestine and liver, and exhibits distinctive developmental and metabolic regulation [9], mediated via the expression and stoichiometric interactions of two dominant *trans*-acting proteins, Apobec-1 and Apobec-1 complementation factor (ACF), although other proteins are implicated [9-12].

Although much is known about the regulation and functional consequences of apoB mRNA editing, remarkably little is known about the range of other targets of C-to-U RNA editing. A recent transcriptome-wide analysis of mouse enterocytes identified 32 novel (non-apoB) Apobec-1-dependent editing targets, all within 3′ untranslated regions (3′ UTRs) [13]. These newly identified RNA targets share features with apoB RNA, including a preference for cytidines embedded in AU-rich regions along with variations of a downstream 11-nucleotide cassette referred to as a 'mooring sequence' with the consensus sequence WRAUYANUAU [13,14]. Those findings raise the corollary questions of whether any of the novel editing targets identified in mouse intestine are also modified in other tissues expressing Apobec-1, particularly the liver, and, if so, are they modified at the same site and to the same extent, and do these editing events lead to differences in mRNA or protein levels?

**Figure 1:**
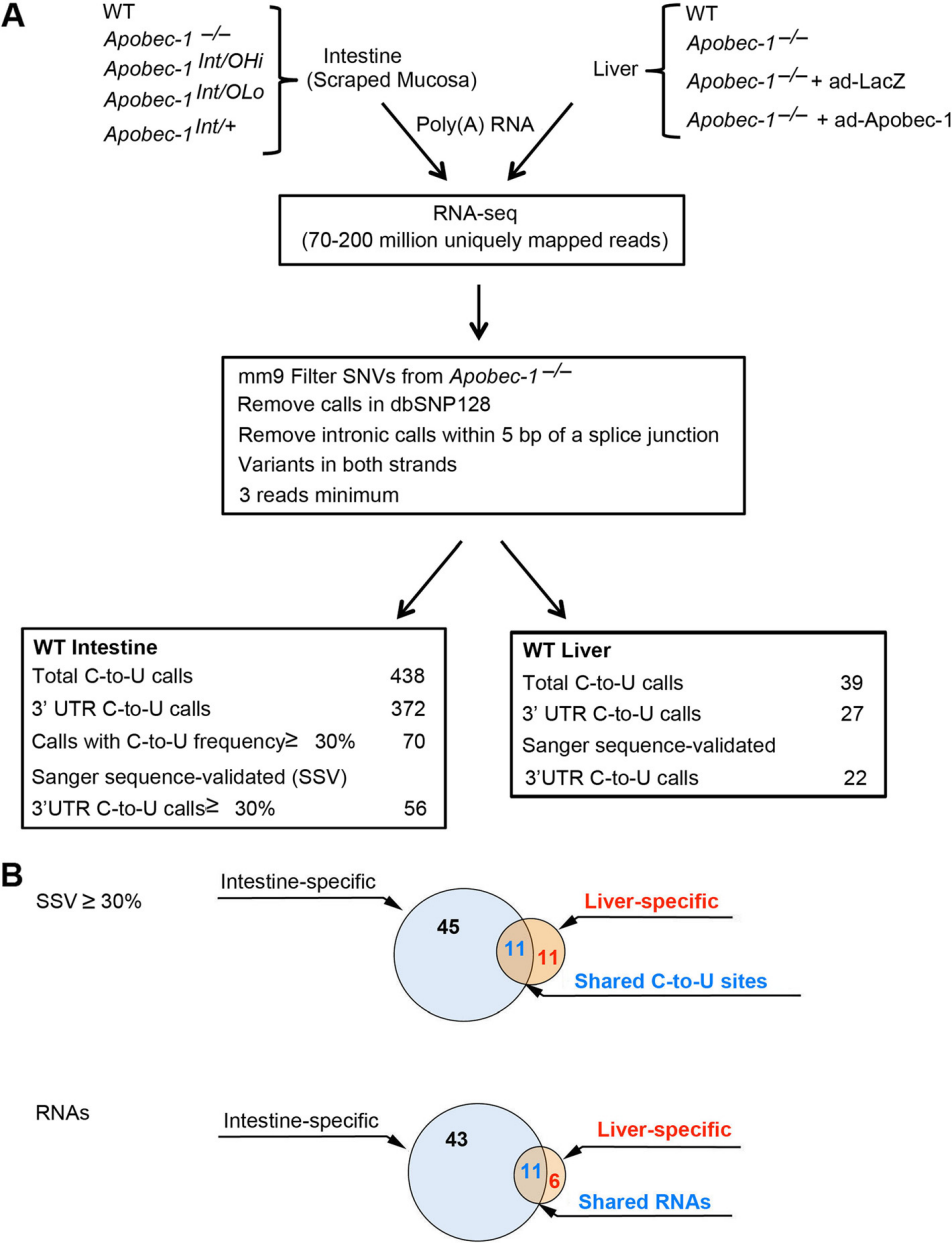
**RNA-seq identification of Apobec-1-dependent RNA-editing targets. (A)** RNA-seq procedure and analyses of 3' UTR C-to-U calls identified in wild-type (WT) small intestine and liver. Five murine lines with distinctive Apobec-1 expression profiles were used for intestinal transcriptome analysis. *Apobec-1*^-*/*-^ mice exhibit no intestinal or hepatic apoB RNA editing. *Apobec-1*^*Int/+*^, intestine-specific Apobec-1 transgenic mice [15], were crossed with *Apobec-1*^-*/*-^ mice generating *Apobec-1*^*Int/OHi*^ and *Apobec-1*^*Int/OLo*^ transgenic mice, with high (Hi) and low (Lo) levels of Apobec-1 expression [15]. WT hepatic transcriptomes were compared to *Apobec-1*^-*/*-^ mice. *Apobec-1*^-*/*-^ + ad-Apobec-1 or ad-LacZ indicates *Apobec-1*^-*/*-^ mice injected with adenovirus expressing Apobec-1 or Lac Z. Overexpression of Apobec-1 in the liver restores apoB RNA editing. Uniquely mapped reads were aligned to the C57BL/6 mouse genome (NCBI37/mm9) containing 23,334 reference genes. To minimize false positive calls, sites identified in both WT and *Apobec-1*^-*/*-^ mice, known SNPs from dbSNP128 and sites lying outside the gene boundaries were excluded. The remaining sites were corrected for strand sense and qualified when supported by 3 minimum non-identical reads, a minimum frequency of 10% with a minimum coverage of 10 reads. An arbitrary cutoff of 30% editing frequency was set to sequence-validate calls identified in the intestine. Due to the low number of calls identified in WT liver, all calls (27) were sequenced. **(B)** Numbers of C-to-U editing events and RNAs Sanger-sequence-validated (SSV). Blue circles represent the 56 3' UTR C-to-U calls identified in 54 WT intestine RNAs. Red circles show the 22 validated C-to-U sites identified in 17 hepatic RNAs. The shaded regions represent the 11 C-to-U sites or RNAs identified in both small intestine and liver. Forty-five sites were specific to the intestine, 11 were liver-specific.

Here we used stringent filtering and sequence validation to reveal multiple new sites of Apobec-1-dependent C-to-U RNA editing, with examples of both tissue-specific and common targets (Figure 1). We show that RNA editing led to corresponding changes in mRNA and protein expression in a subset of mRNAs. We also find enrichment in the edited forms of certain mRNAs in cytoplasmic compared to nuclear fractions. We further show that mRNA editing regulates polysome distribution of a subset of targets. We demonstrate editing of some but not all novel targets using cell-free extracts from wild-type (WT) but not *Apobec-1*-deficient mice, demonstrating that Apobec-1 is necessary for RNA editing. Taken together, our findings demonstrate that C-to-U RNA editing exerts distinct tissue-specific consequences, including a spectrum of outcomes on protein expression.

## Results

### Overview

We undertook a comprehensive comparison of Apobec-1-dependent C-to-U RNA editing, starting with transcriptome-wide analyses of small intestine mucosa and liver from WT and *Apobec-1*^-*/*-^ mice (Figure 1). We then extended those analyses to other lines of mice with low or high transgenic intestinal overexpression of Apobec-1 in either an *Apobec-1*^-*/*-^ (that is, *Apobec-1*^*Int/O*^) or WT background (*Apobec-1*^*Int/+*^) [15] (Figure 1). We further studied livers from *Apobec-1*^-*/*-^ mice following adenoviral delivery of Apobec-1 (ad-Apobec-1) or a LacZ control virus (Figure 1). This strategy permitted an evaluation of tissue-specific (that is, intestine versus liver) and dose-dependent (that is, WT versus *Apobec-1*^*Int/+*^and *Apobec-1*^*Int/OLo*^ versus *Apobec-1*^*Int/OHi*^) changes in C-to-U RNA editing at different levels of Apobec-1 expression [15].

### Identification of novel intestinal and hepatic Apobec-1-dependent editing targets

The first task was to examine the 70 to 200 million RNA-seq reads for intestine and liver from WT and *Apobec-1*^-*/*-^ mice identifying mRNA sequences with C-to-U differences. C-to-U mismatches found in both WT and *Apobec-1*^-*/*-^ mice as well as sites with less than three reads were excluded from further analysis. Results for WT intestine revealed a total of 438 putative editing sites (including apoB), 372 (85%) of which were located in the 3′ UTR, and with the remainder residing in 5′ UTR (7; 1.6%), exonic (7; 1.6%) or intergenic regions (52; 12%). We selected an arbitrary cutoff of 30% C-to-U editing in 3' UTR calls and then validated 56 of 70 calls (80% true positive) in 54 RNAs (App mRNA was edited at two sites) (Tables 1 and 2) by Sanger sequencing, including cohort validation of a subset of 23 of the 31 RNA targets identified by Rosenberg *et al*. [16] (74% true positive) (Table 1). Of the seven exonic sites (six RNAs), two were in apoB (one novel), two others were previously unreferenced SNPs, and the remaining three were false positives based on Sanger sequencing (Table S1A in Additional file 1). C-to-U RNA editing efficiency among the novel 3′ UTR targets ranged from 31 to 84% (Table 2). Together the results (Tables 1 and 2) identify 54 validated Apobec-1-dependent RNA editing targets from mouse intestine, 32 of which have not been reported previously.

**Table 1:** Validation cohort of 3’ UTR Apobec-1 RNA targets

					**Rosenberg *et al*.**[16]			**Current**		
	**RNA**	**Chr**	**Position**	**Reference base**	**Editing frequency**	**Sanger**	**RNA-seq**	**Reads**	**Sanger**	**Edited/total**
1.	Sult1d1	5	87984364 (-)	G	79%	82%	92%	26	91%	(20/22)
2.	Mfsd7b	1	192830761 (-)	G	78%		78%	148	50%	(10/20)
3.	Aldh6a1	12	85772761 (-)	G	56%		68%	38	50%	(10/20)
4.	Usp25	16	77116537 (+)	C	50%		68%	44	58%	(11/19)
5.	Serinc1	10	57235791 (-)	G	75%		66%	50	60%	(12/20)
6.	Tmem30a	9	79617629 (-)	G	55%		65%	29	61%	(11/18)
7.	Bche	3	73442586 (-)	G	36%		61%	52	64%	(14/22)
8.	2010106E10Rik	X	109671648 (+)	C	46%		61%	544	54%	(12/22)
9.	Gramd1c	16	43981376 (-)	G	29%		59%	68	68%	(13/19)
10.	Cmtm6	9	114658289 (+)	C	nd		54%	305	75%	(15/20)
11.	BC013529	1	152209582 (-)	G	45%		50%	35	40%	(8/20)
12.	Cyp4v3	8	46391931 (-)	G	38%	36%	49%	3381	29%	(5/17)
13.	Sh3bgrl	X	106355759 (+)	C	30%	20%	43%	70	40%	(8/20)
14.	Clic5	17	44416335 (+)	C	31%		37%	1243	23%	(5/22)
15.	App	16	84954758 (-)	G	21%		34%	221	4%	(1/22)
	App	16	84955113 (-)	G	21%		30%	1539	21%	(4/19)
16.	Hprt1	X	50374459 (+)	C	22%		31%	168	20%	(4/20)
17.	B2m	2	121978638 (+)	C	18%		28%	803	27%	(6/22)
18.	Tmbim6	15	99239051 (+)	C	20%		24%	1145	30%	(6/20)
19.	Rnf128	X	136207009 (+)	C	20%		24%	779	18%	(4/22)
20.	Rrbp1	2	143811725 (-)	G	38%		22%	269	5%	(1/20)
21.	Sep15	3	144259976 (+)	C	54%	15%	14%	738	23%	(5/22)
22.	Ank3	10	69486962 (+)	C	36%		13%	400	5%	(1/21)
23.	Lrrc19	4	94304303 (-)	G	26%		11%	26	4%	(1/22)

(+) Sense strand.

(-) Antisense strand.

**Table 2:** Wild-type intestine 3' UTR Apobec-1 RNA targets (>30% editing efficiency)

	**RNA**	**Chr**	**Position**	**Reference base**	**RNA-seq**	**Reads**	**Sanger**	**Edited/total**
1.	Cd36	5	17288955 (-)	G	84%	44	85%	(17/20)
2.	Reps2	X	158851906 (-)	G	75%	12	50%	(10/20)
3.	Siglec5	7	50614573 (+)	C	72%	43	10%	(2/20)
4.	Fmn1	2	113556683 (+)	C	71%	17	65%	(13/20)
5.	0610010O12Rik	18	36562329 (+)	C	67%	2900	55%	(11/20)
6.	Mcmbp	7	135841366 (-)	G	63%	155	30%	(7/23)
7.	Man2a1	17	65104330 (+)	C	60%	119	15%	(3/20)
8.	Herc2	7	63486942 (+)	C	60%	15	5%	(1/20)
9.	Ddx60	8	64516163 (+)	C	59%	22	30%	(6/20)
10.	Tmem195	12	38308269 (+)	C	56%	466	44%	(11/25)
11.	Mtmr2	9	13610423 (+)	C	53%	19	25%	(5/20)
12.	Cyp2c65	19	39168358 (+)	C	50%	1011	9.5%	(2/21)
13	Cnih	14	47395982 (-)	G	48%	25	59%	(13/22)
14.	Atp11c	X	57477477 (-)	G	46%	13	5%	(1/19)
15.	Sh3bgrl	X	106356686 (+)	C	45%	83	35%	(7/20)
16.	Fgl2	5	20883372 (+)	C	42%	72	33%	(7/21)
17.	Nr1d2	14	19036726 (-)	G	39%	82	25%	(5/20)
18.	Tmem135	7	96290044 (-)	G	39%	28	20%	(4/20)
19.	Slc4a4	5	89668527 (+)	C	38%	21	45%	(9/20)
20.	Dpyd	3	119134696 (+)	C	38%	66	25%	(5/20)
21.	Ttc9c	19	8885447 (-)	G	37%	16	18%	(4/22)
22.	Yes1	5	32989151 (+)	C	36%	22	30%	(6/20)
23.	1110020G09Rik	15	9038469 (+)	C	36%	22	10%	(2/20)
24.	Actr2	11	19963383 (-)	G	35%	52	41%	(9/22)
25.	Kctd12	14	103379573 (-)	G	35%	63	28%	(5/18)
26.	Nr3c1	18	39571801 (-)	G	33%	18	17%	(4/24)
27.	Skil	3	31018375 (+)	C	33%	16	5%	(1/20)
28.	Ccny	18	9315769 (-)	G	32%	25	25%	(5/20)
29.	Rab1	11	20125336 (+)	C	32%	508	15%	(3/20)
30.	mCG_2776	6	8378189 (+)	C	31%	36	20%	(4/20)
31.	Lrba	4	9503468 (+)	C	31%	37	6%	(1/16)
32.	Dek	13	47181166 (-)	G	31%	26	5%	(1/20)

(+) Sense strand.

(-) Antisense strand.

We attempted to account for discordances between our results and those of Rosenberg *et al*. [13] (Table S1B in Additional file 1). In two instances, miscalled bases reflected the spurious mapping of reads with errors to a small region ('island') of otherwise unexpressed paralogs of an unedited expressed gene. In one instance the location of the editing site was within a homopolymeric stretch of six thymidine residues (Table S1B in Additional file 1), known to be vulnerable to nucleotide insertions [17]. Four targets (BC003331, Ptpn3, Rb1 and Abcb7) were below our 30% editing threshold, but Sanger sequencing nevertheless validated these mRNAs as Apobec-1 targets (Table S1C in Additional file 1). Finally, six additional targets were originally identified in isolated enterocytes [13], rather than from mucosal RNA as in the current study. We then investigated whether the cellular origin of the RNAs might account for these discordances. Sites in Casp6 and Atf2 were sequence-validated using isolated enterocyte RNA (Table S1D in Additional file 1). The other four targets were not validated, for reasons that remain to be determined.

Turning to hepatic RNA targets, we identified a total of 39 putative editing sites, of which 27 were located in 3′ UTRs, with the remainder located in 5′ UTR (2; 5%), exonic (6; 15%) and intergenic regions (4; 10%). Because our filtering algorithms indicated fewer putative editing targets in the liver compared to the small intestine, we undertook sequence validation of the entire set and confirmed 22 of 27 3′ UTR sites (81% true positive) distributed across 17 novel RNA targets (Table 3). Of these 17 liver targets, 11 were also verified by sequence analysis in small intestine, and 6 were unique to liver (Table 3 and Figure 1). Of the 11 RNAs edited in both liver and small intestine, all revealed lower levels of editing in liver (Serinc1: 60 to 66% in intestine by Sanger/RNA-seq versus 9.5 to 38% in liver by Sanger/RNA-seq; Cd36: 85 to 84% in intestine by Sanger/RNA-seq versus 23 to 24% in liver by Sanger/RNA-seq). Most of the shared liver-intestine targets (7/11) were below our threshold for RNA-seq, although Sanger sequencing revealed editing ranging from 4 to 32% (Table 3). Of the six putative exonic editing sites, two (apoB and a novel site in BC005561), were Sanger sequence validated, while four, not validated by Sanger sequencing, were considered as false positives (Table S1E in Additional file 1).

**Table 3:** Wild-type liver 3’ UTR Apobec-1 RNA targets

	**RNA**	**Chr**	**Position**	**Reference base**	**RNA-seq**	**Reads**	**Sanger**	**Edited/total**
1.	Serinc1	10	57235791 (-)	G	38%	186	9.5%	(2/21)
2.	Dcn*	10	96980667 (+)	C	30%	104	14%	(3/21)
	Dcn*	10	96980535 (+)	C	14%	370	14%	(3/21)
3.	Cd36	5	17288955 (-)	G	24%	45	23%	(5/21)
4.	Cybb*	X	9012717 (-)	G	23%	13	18%	(4/22)
	Cybb*	X	9012852 (-)	G	23%	13	4.5%	(1/22)
	Cybb*	X	9013390 (-)	G	14%	21	14%	(2/16)
5.	Colec10*	15	54297696 (+)	C	18%	17	5%	(1/20)
	Colec10*	15	54295026 (+)	C	13%	39	5%	(1/20)
6.	Ube2l3*	16	17152203 (-)	G	16%	271	45%	(9/20)
7.	Abcc9*	6	142538042 (-)	G	14%	28	14%	(3/22)
	Abcc9*	6	142538035 (-)	G	11%	52	18%	(4/22)
8.	Aldh6a1	12	85772761 (-)	G	12%	854	14%	(3/22)
9.	Tmem30a	9	79617629 (-)	G	11%	95	8%	(2/23)
10.	Mpeg1*	19	12539179 (+)	C	11%	66	5%	(1/21)
11.	Usp25	16	77116537 (+)	C	BT^a^	12	9%	(2/22)
12.	Sh3bgrl	X	106355759 (+)	C	BT^b,c^	49	17%	(3/18)
13.	Cmtm6	9	114658289 (+)	C	BT^b,c^	147	4%	(1/22)
14.	Sep15	3	144259976 (+)	C	BT^b^	379	19%	(4/19)
15.	Cyp4v3	8	46391931 (-)	G	BT^b^	350	14%	(3/22)
16.	Rnf128	X	136207009 (+)	C	BT^b,c^	260	32%	(7/22)
17.	B2m	2	121978638 (+)	C	BT^b^	8013	9%	(2/21)

BT, below threshold.

^a^Less than three reads supporting C-to-U editing.

^b^Less than 10% C-to-U editing.

^c^Does not have one read per strand.

(+) Sense strand.

(-) Antisense strand.RNAs with asterisk are liver-specific (compare Figure 1).

Remaining RNAs are shared between liver and intestine.

### Sequence context features for C-to-U RNA editing

Prompted by findings that a close or exact match to the mooring sequence in apoB RNA was present in almost every other Apobec-1-dependent editing site [16], we examined the flanking sequence of editing sites identified above for features that might explain why some RNAs are edited at much higher efficiency than others. We found the region flanking edited 3′ UTRs to be significantly more AU-rich than a random set of 3′ UTRs in both intestine and liver (Figure S1A,B in Additional file 1), which was confirmed by examination of a 101-nucleotide region overlapping the edited sites (Figure S1C,D in Additional file 1). Nearest-neighbor nucleotide analysis revealed a strong preference for adenosine and uridine both upstream (-1) and downstream (+1) of the editing site for both intestinal and liver targets (Figure S1E,F in Additional file 1). However, mismatches in the mooring sequence, which are required for apoB RNA editing [14], did not correlate with intestinal target editing efficiency. For example, Rab1 RNA contained a perfect match to the consensus mooring site and demonstrated 32% editing, while Reps2 RNA contained two mismatches yet exhibited 75% editing (Table S2 in Additional file 1). Thus, the immediate sequence context favors Apobec-1-dependent C-to-U RNA editing, but does not distinguish editing targets by tissue type and does not explain the differences in editing efficiency.

### Apobec-1 abundance modulates tissue-specific editing efficiency

Previous work demonstrated that transgenic liver overexpression of Apobec-1 produced additional editing sites (so called ‘hyperediting’) in apoB mRNA and in other targets [18,19]. In order to understand the importance of Apobec-1 expression levels in editing target selection and efficiency, we generated intestinal Apobec-1 transgenic mice on either a WT or *Apobec-1*^-*/*-^ background [15] and compared editing efficiencies at different levels of transgene expression among shared RNAs from the indicated genotypes. Specifically, we compared editing efficiencies of shared targets between WT and *Apobec-1*^*Int/+*^ and editing efficiencies of RNA targets shared between *Apobec-1*^*Int/OLo*^ and *Apobec-1*^*Int/OHi*^. Among Apobec-1-dependent editing targets in WT mice (Table 2), a subset demonstrated increased RNA editing in response to increasing levels of Apobec-1 expression (Table S3A in Additional file 1). For example, ATP6ap2 demonstrated 28 to 30% editing in WT and 57 to 62% with transgenic overexpression (*Apobec-1*^*Int/+*^), but no detectable editing in *Apobec-1*^-*/*-^ mice. Similarly, editing efficiency of ATP6ap2 increased in *Apobec-1*^*Int/Hi*^ versus *Apobec-1*^*Int/Lo*^ mice (Table S3A inAdditional file 1). The fold increase observed was variable among RNAs, ranging from 1.2- (Usp25) to 4-fold (Rab1) in WT versus *Apobec-1*^*Int/+*^ mice and from 3 to 80 fold in *Apobec-1*^*Int/Lo*^ versus *Apobec-1*^*Int/Hi*^ mice. Occasional discordance was found for editing efficiency as inferred from RNA-seq versus Sanger sequencing. For example, Atp6ap2 in *Apobec-1*^*Int/Lo*^ mice demonstrated 52% editing by RNA-seq but only 4.5% by Sanger sequencing (1/22 clones edited). Overall, most but not all RNA targets demonstrated increased editing efficiency with increasing Apobec-1 expression (Table S3A in Additional file 1).

Examination of eight hepatic RNA editing targets identified in both WT and *Apobec-1*^-*/*-^ mice following ad-Apobec-1 transduction revealed increased editing efficiencies for all shared targets from two- (Tmem30a) to nine-fold (Serinc1) (Table S3B in Additional file 1). Additional C-to-U editing sites (hyper-editing) were also detected (Table S3C in Additional file 1); among the eight shared targets, seven RNAs exhibited from one to nine additional editing sites (Table S3C inAdditional file 1). In addition, as noted above, alignment of nucleotides flanking these edited sites revealed a strong preference for A or U immediately upstream (96%) and downstream (92%) of the edited site, respectively (Figure S1F in Additional file 1) and, as noted above, alignment with the mooring sequence failed to reveal a predictive correlation with hepatic editing efficiency (Table S4 in Additional file 1).

### *In vitro* validation of Apobec-1-dependent RNA editing

**Figure 2:**
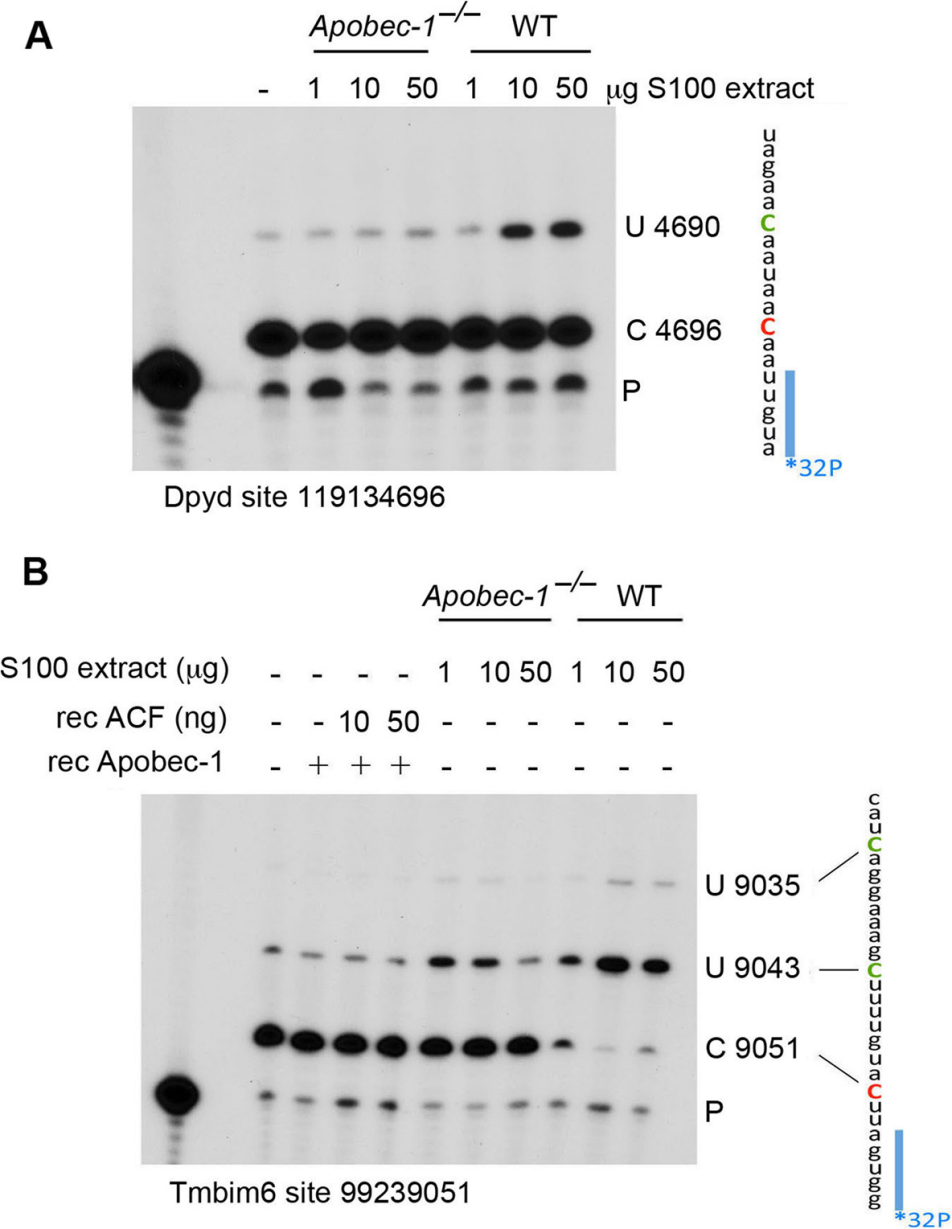
***In vitro *editing assay of 3' UTR targets.** Total hepatic RNA from *Apobec-1*^-*/*-^ mice was incubated with increasing amounts of WT hepatic S100 extract. RNA was used for cDNA synthesis followed by PCR amplification of Apobec-1 3′ UTR targets using specific targets. **(A)** Endogenous Dpyd RNA editing of cytidine 119134696 was determined by poisoned primer extension. The relative mobility of the unedited (C 4696) and edited product (U 4690) is indicated to the right. Vertically is shown the sequence surrounding the editing site. The targeted cytidine is indicated in red. Upon editing, the primer extension reaction proceeds until the next C (represented in green). The ^32^P-labeled primer is shown in blue. **(B)** Endogenous Tmbim6 RNA editing of cytidine 99239051. Total hepatic RNA from *Apobec-1*^-*/*-^ mice was incubated with recombinant Apobec-1 and ACF or with increasing amounts of hepatic WT S100 extract. C-to-U editing of cytidine 9051 was determined by poisoned primer extension. To the right is shown the sequence surrounding the editing site. The edited cytidine (9051) is shown in red. Cytidine 9043 also appears to be targeted, resulting in an extension product terminating at cytidine 9035.

Because C-to-U RNA editing of a synthetic apoB RNA template can be accomplished using recombinant Apobec-1 and ACF, we asked if editing of these novel targets might also be replicated in an *in vitro* system. We used a cell-free *in vitro* editing assay in which RNA from *Apobec-1*^-*/*-^ liver was incubated with tissue S100 extract and analyzed by poisoned primer extension analysis [9]. This strategy was employed on two candidate RNAs, selected based on their prior identification in small intestine [16] and independently in brain [4]. We found that Dpyd was approximately 30% edited (Figure 2A), while Tmbim6 site 99239051 demonstrated almost complete editing with increasing amounts of WT extracts. For both RNAs, editing was absent in extracts prepared from *Apobec-1*^-*/*-^ mice (Figure 2B). C-to-U RNA editing could not be replicated using recombinant Apobec-1 and ACF alone (Figure 2B), conditions previously shown to support *in vitro* RNA editing of apoB [9]. We note that other targets, including Cmtm6, Sh3bgrl, Serinc1 and Cyp4v3, failed to replicate C-to-U editing in this cell-free system (data not shown). Together these findings show that Apobec-1 is required for C-to-U RNA editing and suggest that other factors in addition to ACF may be required for target selectivity and *in vitro* C-to-U deamination.

### Nucleo-cytoplasmic distribution of edited RNAs

**Figure 3:**
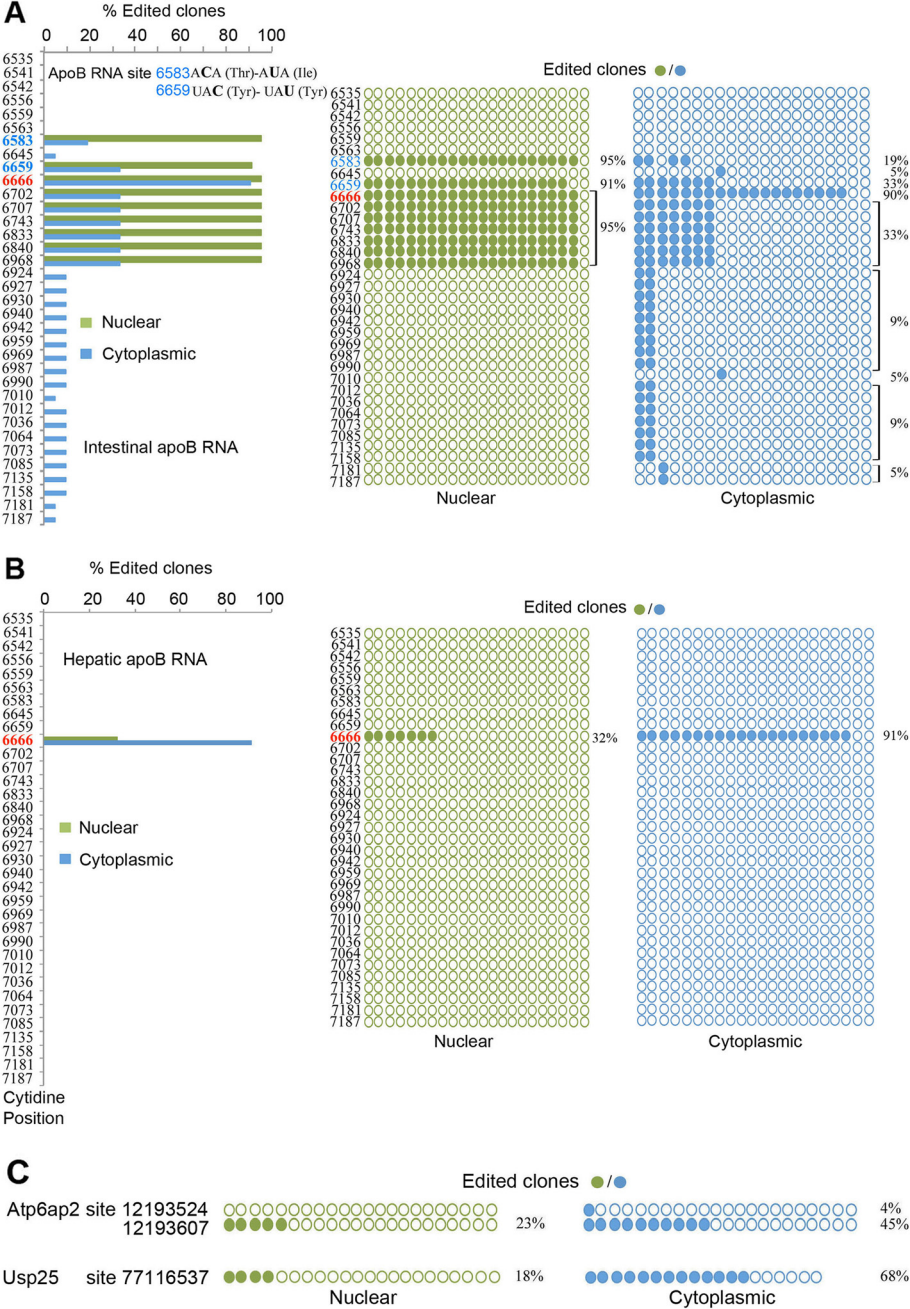
**Nucleo-cytoplasmic distribution of Apobec-1-dependent mRNA editing targets. (A,B)** Distribution of WT small intestine **(A)** and hepatic **(B)** edited apoB RNA. A 738 bp amplicon (nucleotides 6,508 to 7,246) from nuclear and cytoplasmic apoB mRNA was cloned and sequenced. Twenty-two clones from each subcellular fraction (from three independent nuclear-cytoplasmic isolations) were analyzed. Left panel: graphic representation of percentage of edited clones in nuclear and cytoplasmic apoB RNA. Right panel: targeted cytidines identified in nuclear apoB RNA are indicated with green circles; cytidines identified in cytoplasmic apoB RNA are represented by blue circles. All cytidines are aligned with the nucleotide position to the left. **(C)** Nuclear-cytoplasmic distribution of intestinal Apobec-1 3′ UTR targets identified by RNA-seq and validated by Sanger sequencing. A 550 bp (ATP6ap2) and a 667 bp (Usp25) amplicon were generated from nuclear and cytoplasmic RNA and analyzed by sequencing 19 to 22 clones. For both ATP6Ap2 and Usp25 RNAs, the edited RNA is predominantly exported to the cytoplasm.

Earlier studies demonstrated that apoB RNA undergoes post-transcriptional RNA editing in the nucleus of rat liver [20]. Those findings demonstrated that C-to-U RNA editing was virtually complete on spliced, polyadenylated intranuclear apoB RNA and that little if any additional editing took place in the cytoplasmic compartment [20]. We confirmed that >90% intestinal apoB RNA was edited at the canonical site (6666) in both nucleus and cytoplasm, but in addition observed several subpopulations of edited apoB RNAs with distinctive nucleo-cytoplasmic distributions (Figure 3A). Specifically, intestinal nuclear apoB RNA contained a cluster of C-to-U sites distributed between positions 6702 and 6968 in addition to the canonical 6666 site (Figure 3A). None of these sites was edited in liver RNA (Figure 3B). Unexpectedly, intestinal nuclear apoB RNA also demonstrated extensive (>90%) exonic C-to-U editing at positions 6583 and 6659. These sites were again not edited in liver RNA (Figure 3B). RNA editing at position 6583 modifies an A*C*A to an A*U*A codon, resulting in a threonine to isoleucine substitution, while editing at position 6659 (UA*C* to UA*U*) is a silent modification (Tyr-Tyr) (Figure 3A). In addition, a much lower proportion (19 to 33%) of cytoplasmic apoB RNA contained these two additional edited exonic sites (6583, 6659) compared with what was observed (approximately 90%) in the nucleus.We next turned to the nucleo-cytoplasmic distribution for other editing targets. For Atp6ap2, we identified two edited sites (positions 12193607 and 12193524; Figure 3C). Atp6ap2 RNA edited at both sites was detected only in the cytoplasm and at low frequency (4%, 1/22 clones edited). By contrast, Atp6ap2 RNA containing only the edited site 12193607 was abundantly represented in cytoplasm (45%, 10/22 edited clones) compared to nucleus (23%, 5/22 edited clones sequenced). For Usp25, we identified only a single RNA population edited at site 77116537 and found 68% of the transcripts containing the edited site in cytoplasm (13/19 edited clones) but only 18% editing in nuclear transcripts (4/22 edited clones). Among the testable hypotheses to account for these observations is that RNA editing of Atp6ap2 and Usp25 may favor cytoplasmic export or influence the pathways modulating turnover of the edited RNA. The extent to which other edited RNAs show differences in subcellular distribution remains to be determined.

### Apobec-1-mediated changes in mRNA abundance and microRNA seed sites

**Figure 4:**
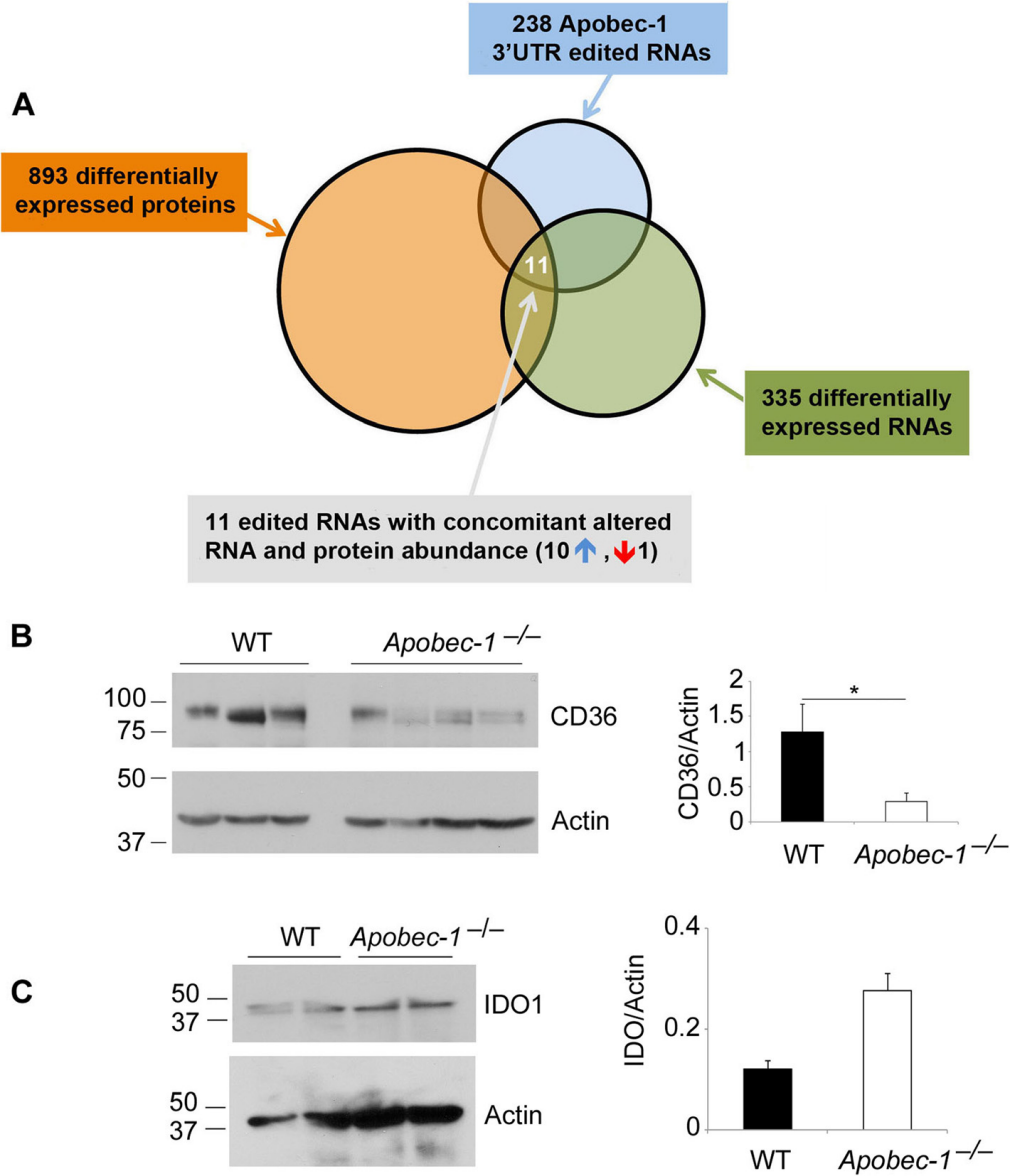
**Apobec-1 editing targets in relation to RNA and protein expression. (A)** Schematic representation of Apobec-1-dependent editing targets in relation to RNA and protein expression. Total proteins were extracted from WT *Apobec-1*^-*/*-^ intestine and submitted for proteomic analysis (Materials and methods). The relative expression and editing status of the RNAs encoding the differentially expressed proteins were analyzed in parallel. Data comparison between WT and *Apobec-1*^-*/*-^ data sets revealed 238 Apobec-1 RNA editing targets (blue circle), 335 differentially expressed RNAs (green circle) and 893 differentially expressed proteins (orange circle). Overlapping these three groups led to the identification of only 11 edited RNAs showing altered expression concomitant with altered protein level: 10 RNAs and proteins were up-regulated in WT (blue upward arrow) and one RNA and its protein product were down-regulated in WT compared to *Apobec-1*^-*/*-^ (red downward arrow). **(B)** Reduced expression of Cd36 in intestinal extracts from *Apobec-1*^-*/*-^ mice. Total cell lysates from three individual WT mice and four individual *Apobec-1*^-*/*-^ animals were separated by SDS-PAGE probed with an anti-Cd36 and anti-α-actin antibody. * Indicates p **<** 0.05 for difference in protein abundance **(C)** Trend to increased expression of Ido1 protein expression in western blots of intestinal extracts from two individual *Apobec-1*^-*/*-^ mice and two individual WT mice, normalized to α-actin as a loading control. Error bars represent mean ± se of relative protein abundance by genotype.

We next asked whether RNA editing exerts functional effects on the modified transcripts. We undertook transcriptome-wide comparison of intestinal mRNA abundance of the 58 validated editing targets, of which 32 derived from Table 2, 22 from Table 1, and 4 from Table S1B in Additional file 1. The data show that approximately half (27) were significantly down-regulated in *Apobec-1*^-*/*-^ intestine (lower FPKM (fragments per kilobase of exon per million), as inferred from RNA-seq alignment frequency; see Materials and methods) and a subset of these same samples were validated with quantitative PCR (Table S5 in Additional file 1). The remainder showed either no change or (in a single case, Dek) a trend to increased mRNA abundance (more than two-fold) in *Apobec-1*^-*/*-^ mice (Table S5 in Additional file 1). Similar analysis of liver RNA revealed one target (Cd36) down-regulated (more than two-fold) in *Apobec-1*^-*/*-^, but the majority of targets (11/16) showed no change in expression (Table S6 in Additional file 1). Among the 335 differentially expressed mRNAs (Figure 4A), a subset of 17 demonstrated C-to-U RNA editing, although there was no correlation between the extent of editing and mRNA abundance (Figure S2A,B in Additional file 1).

Several studies show that A-to-I RNA editing modifies microRNA (miRNA) sites and influences mRNA abundance [5,21,22]. Accordingly, we investigated the possibility that C-to-U editing might create, eliminate or change the affinity of miRNA seed sequences that in turn might influence gene expression. For intestinal targets, the Siglec 5 editing site is contained within four miRNA seed motifs (Table S7 in Additional file 1). Interestingly, loss of Siglec5 RNA editing in *Apobec-1*-deficient mice resulted in a nine-fold decrease in mRNA abundance (Table S5 in Additional file 1) and not only eliminates four of those miRNA sites (from WT mice), but simultaneously creates five new seed motifs (Table S7 in Additional file 1). By contrast, C-to-U editing creates miRNA seed motifs in five other RNA targets (App, Cnih, B2m, Mtmr2 and Sh3bgrl) that show no change in mRNA expression (Table S7 in Additional file 1). For liver samples, loss of CD36 editing in *Apobec-1*^*-/-*^ mice led to a two-fold mRNA decrease compared to WT samples, yet simultaneously eliminated a miRNA seed motif (Table S8 in Additional file 1). Furthermore, RNA editing created miRNA seed motifs in three other hepatic targets whose mRNA abundance either increased in *Apobec-1*^-*/*-^ mice or remained unchanged (Table S8 in Additional file 1). Taken together, the findings reveal no consensus mechanism by which C-to-U editing within the 3′ UTR alters miRNA binding sites and influences mRNA abundance.

### Apobec-1-dependent C-to-U RNA editing influences protein abundance

Since we did not observe a consensus mechanism by which RNA editing regulates mRNA abundance, we asked if RNA editing might influence translational efficiency. We turned to a proteome-wide approach using mass spectrometry-based shotgun proteomics in conjunction with metabolic labeling for quantification to identify 893 proteins that were differentially expressed in small intestine from WT versus *Apobec-1*^-*/*-^ mice (Table S9 in Additional file 1). Comparison with our transcriptome-wide analyses revealed 26 differentially expressed proteins encoded by an RNA target of Apobec-1 dependent C-to-U editing (Figure 4A; Table S10 in Additional file 1). Using a two-fold change in protein expression as a cutoff, we demonstrated a concordant increase in both mRNA and protein expression in WT compared to *Apobec-1*-deficient mice in 10 targets (Table S10 in Additional file 1). One additional target (Ido1) showed a decrease in both RNA and protein abundance in WT compared to *Apobec-1*-deficient mice (Tables S10 and S11 in Additional file 1). We confirmed this pattern of differential intestinal protein expression for two targets, Cd36 (which showed the greatest magnitude of C-to-U RNA editing, 84%) and Ido1 (Figure 4B,C). Cd36 RNA was demonstrated to be approximately two-fold down-regulated in *Apobec-1*^-*/*-^ intestine (FPKM and quantitative PCR) (Table S5 in Additional file 1). Western blot analysis showed an approximately four-fold decrease of Cd36 protein expression in *Apobec-1*^-*/*-^ intestine (Figure 4B). Analysis of Ido1 revealed a trend towards increased protein expression in *Apobec-1*^-*/*-^ intestine, consistent with the findings from the proteomic survey (Figure 4C).

**Figure 5:**
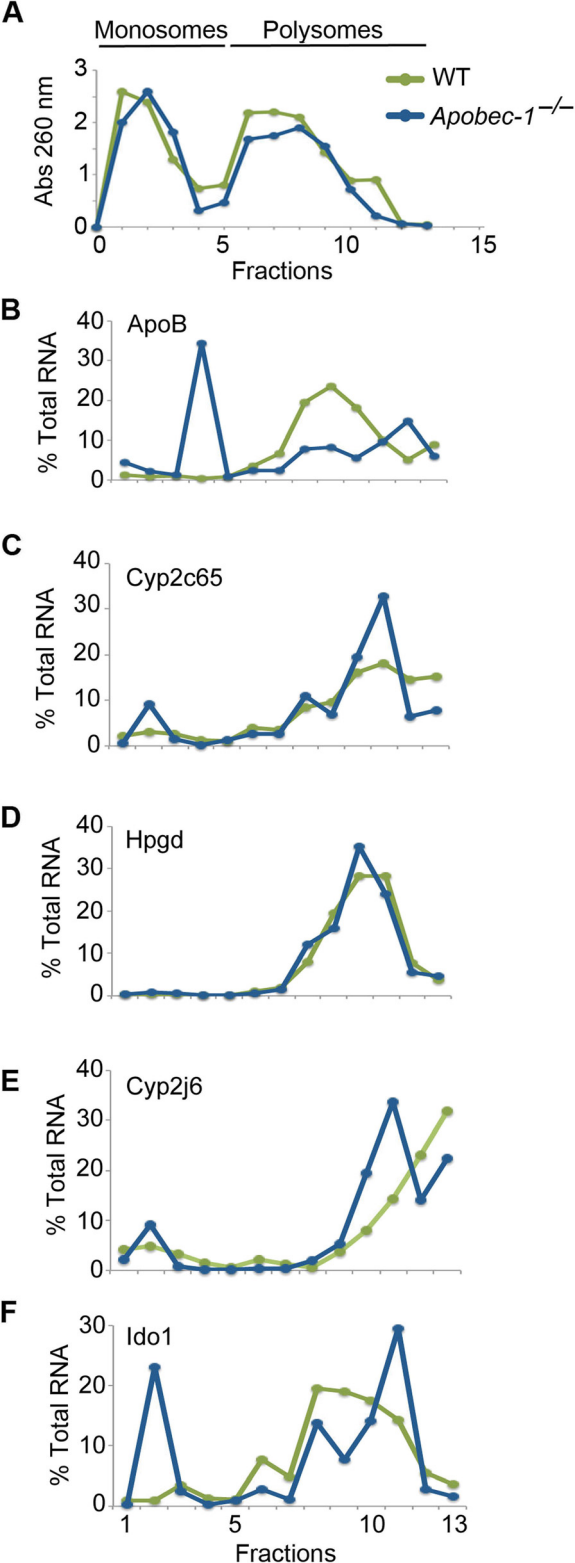
**Polysomal distribution of Apobec-1 mRNA editing targets. (A)** Absorbance profile (A_260_) of fractions harvested from WT (green) and *Apobec-1*^-*/*-^ (blue) mouse small intestine cytoplasmic extracts separated on sucrose gradients. Cytoplasmic extracts (two to five preparations) were prepared, each with three to four animals per genotype. **(B)** Sucrose gradient fractionation of apoB RNA from WT (green) and *Apobec-1*^-*/*-^ small intestine cytoplasmic extracts (blue). ApoB RNA content in each fraction was analyzed in triplicate by quantitative PCR. Data were normalized to the expression of 18S mRNA and expressed as percentage of total apoB RNA. Data represent the mean of four to five separate isolations. **(C-F)** Polysomal distributions of Cyp2c65, Hpgd, Cyp2j6 and Ido1 RNAs, respectively, evaluated by quantitative PCR as described above. WT distribution (green), *Apobec-1*^-*/*-^ distribution (blue).

In seeking an explanation for the changes in protein expression, we considered the possibility that RNA editing influenced mRNA translation by shifting transcript distribution within translating ribosome subfractions. WT intestinal extracts revealed 95% apoB RNA segregated into polysomal fractions while apoB RNA from *Apobec-1*-deficient mice was distributed into both polysome and monosome fractions (Figure 5A,B). We extended this analysis to editing targets that demonstrated alterations in both mRNA and protein abundance. Cyp2c65 RNA from WT mice fractionated predominantly into polysomes but *Apobec-1*-deficient mice showed distinctive populations of RNA associated with monosome fractions (Figure 5C). By contrast, intestinal Hpgd mRNA revealed virtually overlapping profiles in WT and *Apobec-1*^-*/*-^ mice (Figure 5D). Intestinal Cyp2j6 mRNA associated mostly with high molecular weight polysome fractions in WT animals but revealed a shift into lighter fractions in *Apobec-1*^-*/*-^ mice (Figure 5E). Intestinal Ido1 mRNA demonstrated a shift into monosome-associated fractions in *Apobec-1*^-*/*-^ mice (Figure 5F). These findings together suggest that Apobec-1 and C-to-U RNA editing individually influence polysome loading of a subset of target RNAs (including apoB), and (with the exception of Ido1 whose protein abundance was increased in *Apobec-1*-deficient mice) suggest a plausible mechanism whereby editing might selectively influence protein expression (Table S10 in Additional file 1).

## Discussion and conclusions

Here we report a comprehensive, comparative analysis of Apobec-1-dependent C-to-U RNA editing in mouse intestine and liver and show that the functional effects are both transcript- and tissue-specific. These tissues were selected because they represent the dominant sites of expression of both Apobec-1 as well as its canonical target, apoB. Our approach included Sanger sequence validation to reinforce the confidence of the findings (74 to 81% true positive), an important consideration in view of recent transcriptome-wide analyses reporting approximately 49% false discovery rates for non A-to-I RNA editing [5]. Given that we restricted our analysis to 3′ UTR targets and for the small intestine to targets showing at least 30% C-to-U RNA editing, the findings represent a conservative view of the scale of Apobec-1-dependent C-to-U RNA editing and its functional implications.

We validated most but not all the findings of transcriptome-wide Apobec-1-mediated RNA editing in enterocytes [16]. Some of the discordances were accounted for by differences in the optimized parameters [23] used in the current report, including filters for sequence quality, strand bias, distance to end of reads, paired-end reads and genomic single nucleotide variants. But it remains possible that other, cell-specific events, including nutritional or circadian factors, might contribute to the differences noted. In addition, the current findings show a restricted subset of shared RNA editing targets between intestine and liver. Other work showed 25% overlap in RNA editing targets in mouse liver and adipose [2], while another study found approximately 53 to 61% concordance in RNA editing (overwhelmingly A-to-I) in seven mouse tissues (including brain and liver but not small intestine) [4]. Nevertheless, among those studies and in the present report, there was conservation in the editing sites identified within each target. The demonstration of fewer C-to-U editing targets in the liver (27) compared to small intestine (372), as well as the reduced range of hepatic (<45%) versus intestinal (approximately 85%) editing efficiency, further emphasize tissue-specific requirements for target selection and cytidine deamination by the hepatic editing machinery. In keeping with this suggestion, only a single edited site was detected for apoB RNA editing in both nuclear and cytosolic hepatic RNA, compared to eight additional sites in intestinal apoB.

Examination of nuclear and cytoplasmic RNA targets revealed unanticipated results. We found that nuclear apoB RNA from WT intestine (but not liver) exhibited extensive C-to-U editing at two exonic sites upstream of the canonical site 6666, one of which (6583) introduces a threonine to isoleucine coding change. There were additional RNA editing sites in nuclear apoB RNA, predominantly 3′ of the canonical site, which were detectable at much lower levels in cytoplasmic RNA. These findings suggest nuclear transcriptomes are relatively enriched with C-to-U edited targets, as suggested recently for A-to-I RNA editing [22]. Among the possibilities to account for the observed differences in nuclear versus cytoplasmic distribution and efficiency of apoB RNA editing, it is tempting to speculate that nuclear apoB transcripts edited at the canonical site may be preferentially exported to the cytoplasm and/or that C-to-U RNA editing influences nucleo-cytoplasmic transport of apoB RNA in a site-specific manner. These possibilities will require formal evaluation in future studies. In this regard, it is worth noting that both Apobec-1 and its RNA binding cofactor ACF have been shown to shuttle between nuclear and cytoplasmic compartments [24,25]. In addition, it should be emphasized that the physiological relevance of compartmentalization of editing targets remains unresolved, with some studies showing A-to-I edited RNAs to be retained in the nucleus [26] while others found A-to-I edited mRNAs preferentially distributed in cytoplasmic translating polysome fractions [27].

The finding that editing sites were concentrated in 3′ UTRs suggests a regulatory role in the transport, stability, translation or other function of these targeted RNAs. Elimination of A-to-I RNA editing in ADAR-null flies resulted in upregulation of hundreds of RNAs [28]. By contrast, we found that mRNA abundance of the majority of edited mRNAs was either unchanged or decreased in *Apobec-1*-deficient mouse intestine. In addition, while other work has demonstrated ADAR-mediated editing of both miRNAs and mRNAs [21], we found no evidence for C-to-U editing of miRNAs from WT small intestine (data not shown). That said, it is possible that either Apobec-1 binding and/or editing affect the stability of the target mRNA-polysome complexes and selectively modulates translational efficiency. For example, RNAs bound to a subset of yeast RNA binding proteins interact with RNA recognition elements located in the 3′ UTR that, in turn, regulate translation [29]. It is worth noting that the 26 differentially expressed proteins encoded by Apobec-1 RNA targets (Table S10 in Additional file 1) represent approximately 3% of the 893 differentially expressed proteins (Figure 4A). By contrast, the 54 Apobec-1 C-to-U RNA editing targets identified by RNA-seq (Tables 1 and 2) represent approximately 1.7% of the total proteins identified in our proteomic survey (Materials and methods), suggesting a two-fold enrichment of proteins encoded by Apobec-1 RNA targets within the pool of differentially expressed proteins (*P*-value 0.0163).

The search to understand the functional implications of RNA editing led to another intriguing observation: a subset of 10 targets exhibited downregulation of both mRNA and protein abundance while a single edited target, Ido1, was upregulated in *Apobec-1*-deficient intestine. We confirmed that another highly edited intestinal Apobec-1-dependent target, Cd36, also showed concordant decreases in RNA and protein abundance in *Apobec-1* null mice. The functional implications of these changes will require formal confirmation but targets including Rfk, Tes, Pde5a, Yme1l1 and Ido1 have been implicated in tumorigenesis [30-35]. This possibility is intriguing in view of findings that *Apobec-1* deletion attenuates the tumor burden in *Apc*^*Min/+*^ mice [36] while deficiency of Deadend1 (*Dnd1*), a paralog of ACF, increases intestinal polyposis susceptibility in *Apc*^*Min/+*^ mice [37]. Among the down-regulated targets in *Apobec-1*-deficient intestine, Cyp3a11, Cyp2c65, Abcd3, Cyp4v3 and Pde5a are either directly or indirectly modulated by lipid mediators and it is possible that the changes observed are a secondary consequence of alterations in lipid flux rather than a direct effect of eliminating RNA editing [35,38-40]. The consequences for intestinal lipid metabolism of the changes in the fatty acid translocase Cd36 [41] are particularly intriguing and will be the focus of future investigation. Alternatively, and by analogy to events described with ADAR-mediated RNA editing, it is conceivable that the changes in protein expression in targets undergoing Apobec-1-dependent C-to-U RNA editing could reflect subtle protein-RNA interactions that influence polysome distribution and in turn modulate gene expression [42]. The current findings demonstrate that Apobec-1-dependent C-to-U RNA editing exerts broad functional effects in a tissue-specific manner, beyond its canonical target apoB and in most cases unrelated to a restricted role in chylomicron assembly.

## Materials and methods

### Animals

All studies were performed using C57BL/6 from JAX (C57BL/6J) or *Apobec-1*^-*/*-^ mice (both genders) backcrossed for >12 generations onto a C57BL/6 background. *Apobec-1*^*Int/O*^ mice and intestinal Apobec-1 transgenic mice [15] were on a mixed background (C57BL/6 and 6xCBA). *Apobec-1*^-*/*-^ mice were injected with 6 × 10^8^ plaque-forming units of recombinant adenovirus encoding either β-galactosidase (Lac-Z) or rat Apobec-1 (ad-Apobec-1) resulting in hepatic Apobec-1 overexpression. Mice were 8 to 10 weeks old and fed an *ad libitum* chow diet. All animals were treated following National Institutes of Health guidelines and all protocols (#20130037) were approved by the Washington University Institutional Animal Care and Use Committee.

### Accession numbers

RNA sequencing data from deep sequencing are available in the Gene Expression Omnibus under the accession number [GEO:GSE57910]. The mass spectrometry proteomics data have been deposited to the ProteomeXchange Consortium [43] via the PRIDE partner repository [44] with the dataset identifier PXD001007.

### RNA-seq library

Total RNA was extracted from intestinal mucosa from WT, *Apobec-1*^-*/*-^ and *Apobec-1*^*Int/O*^ mice and from livers isolated from WT, *Apobec-1*^-*/*-^*, Apobec-1*^-*/*-^ + ad-LacZ and *Apobec-1*^-*/*-^ + ad-Apobec-1 mice (three mice per genotype), using TRIZol reagent (Invitrogen, Grand Island, NY, USA). DNAse-free RNAs were used for cDNA preparation as previously described [6]. Pooled RNA (10 μg) was subjected to oligo(dT) selection. After chemical fragmentation RNA was reverse transcribed using random hexamer and sequencing adapters (Illumina) ligated to each end of double-stranded cDNA. The fragments were then PCR-amplified using linker-specific primers (Illumina). All libraries were diluted to 10 nM and an equal volume of each sample was combined to form the final sequencing pool that was run on an Illumina HiSeq2000.

### RNA-seq analysis

RNA-seq reads for each genotype were mapped to the mouse reference genome (NCBI37/mm9) and single nucleotide variants were called using a modified version from [23]. Reads from each sample were mapped with Bowtie version 0.12.8, with at most three mismatches, suppressing all alignments for a particular read if more than one reportable alignment exist for it, and using only those alignments that fell into the best stratum The alignment files were sorted and indexed using Samtools version 0.1.18 [45]. Variants were called using the mpileup command. We called a single nucleotide variant when at least three independent reads support a non-reference variant, and the variant is present at a minimum frequency of 10% with minimum coverage of 10 reads and is supported by at least one read per strand. Sites were removed if they had three or more different observed nucleotide variants and a minimum frequency greater than 1.5%. Editing candidate sites were required to have no more than a 5% variant frequency in *Apobec-1* knockout genotypes. Known SNPs from dbSNP128 that were not annotated as based on cDNA and sites lying outside of the 5′ and 3′ gene boundaries were set aside, and the remaining sites were corrected for strand sense. These sites were then annotated using ANNOVAR [46] with a splicing threshold of 5.

### Sanger sequencing validation of Apobec-1-dependent editing sites

Genomic DNA and total RNA were isolated from intestine and liver of WT, *Apobec-1*^-*/*-^, *Apobec-1*^*Int/O*^ and *Apobec-1*^-*/*-^ + ad-Apobec-1 mice. Genomic DNA was prepared as follows: 100 ng of liver tissue was incubated at 55°C overnight in 600 μl cell lysis solution (QIAGEN, Valencia, CA, USA). After protein removal, DNA was precipitated and resuspended. Total RNA was TRIzol-extracted and subjected to cDNA synthesis using random hexamers and MultiScribe Reverse Transcriptase from High Capacity cDNA Reverse Transcription kit (Applied Biosystems, Foster city, CA, USA). Both isolated genomic DNA and cDNA were used as templates to amplify sequences containing RNA-seq-identified Apobec-1-dependent putative editing sites. PCR amplifications were performed using Pfultra II DNA polymerase (Agilent Technologies, Santa Clara, CA, USA). Primer sequences are listed in Tables S13 and S14 in Additional file 1. Quality-controlled PCR products were then cloned into pCR-Blunt II-TOPO vector (Invitrogen) following the manufacturer’s recommendations. Twenty individual clones were sequenced using Applied Biosystems BigDye terminator mix version 3.1. C-to-U calls are referred to as true positives when validated by Sanger sequencing. By contrast, C-to-U calls made from RNA-seq but not verified by Sanger sequencing are referred to as false positives.

### Nuclear-cytoplasmic RNA isolation

Intestines were harvested from three to four mice per genotype. Preparation of nuclear and cytoplasmic RNAs was undertaken as described [47]. Briefly, scraped intestinal mucosa was resuspended in ice-cold buffer B (10 mM tris pH 7.4, 140 mM NaCl, 1.5 mM MgCl_2_, 0.5% NP-40, 1 mM DTT, 20 units/μl RNAse inhibitor (Promega Madison, WI, USA) and 1× protease inhibitor) homogenized and centrifuged at 7,000 g for 10 minutes at 4°C. Supernatant was saved as cytoplasmic fraction. Nuclear pellets were resuspended in 2 volumes of buffer B supplemented with one- tenth volume detergent (3.5% sodium deoxycholate (w/v) and 6.6% Tween 20 (v/v)) incubated for 30 minutes at 4°C and centrifuged at 1,000 g for 5 minutes. Supernatant was combined with the previous cytoplasmic fraction and nuclear pellet was rinsed once in buffer B. Cytoplasmic and nuclear RNAs were extracted using TRiZol (Invitrogen) following the manufacturer’s protocol, treated with DNAse (Ambion Life Technology, Grand Island, NY, USA) and subjected to cDNA synthesis as described above. Targets of interest (apoB, Usp25 and ATP6ap2) were then PCR amplified using specific primers (Table S13 in Additional file 1). PCR products were cloned and sequenced as described above.

### Protein extraction and western blotting

Scraped mucosa was homogenized in tissue lysis buffer containing 20 mM Tris (pH 8), 0.15 M NaCl, 2 mM EDTA, 1 mM sodium vanadate, 0.1 M sodium fluoride, 50 mM β-glycerophosphate, 5% glycerol, 2× protease inhibitor (Roche Applied Science Indianapolis, IN, USA), 1% Triton, and 0.1% SDS. Aliquots of homogenate (60 μg protein) were resolved by SDS-PAGE, transferred to PVDF membrane, and probed with goat anti-CD36 antibody (AF2519, R&D Minneapolis, MN, USA), mouse anti-IDO1 (BioLegend, San Diego, CA, USA). Equal loading was verified using a rabbit anti-α-actin antibody (Sigma-Aldrich St. Louis, MO, USA).

### Polysome isolation

Each polysome isolation used three to four mice with two to five isolations per genotype. Intestinal mucosa was prepared in ice-cold phosphate-buffered saline supplemented with 100 μg/ml cyclohexamine (Sigma, St Louis, MO, USA) was incubated in 1 ml lysis buffer (25 mM Tris-HCl pH 7.5, 250 mM NaCl, 5 mM MgCl_2_, 0.5 mM PMSF, 200 μg/ml heparin (Sigma), 5 mM dithiothreitol, 20 U/ml RNAsin, 100 μg/ml cycloheximide, 1% Triton X-100, 1× protease inhibitor). Scraped mucosa was homogenized and centrifuged at 10,000 g for 10 minutes at 4°C. The supernatant was loaded onto a 10 to 50% sucrose gradient and centrifuged at 40,000 rpm for 2.25 h at 4°C using an SWT41i rotor (Beckman Brea, CA, USA). Fractions (900 μl) were collected from the bottom of the gradient and 260 nm absorbance monitored by spectrophotometry. RNA was phenol/chloroform extracted from each fraction, precipitated, resuspended in 20 μl H_2_O and used for cDNA synthesis followed by PCR amplification of specific targets (apoB, Usp25, Atp6ap2). PCR products were cloned and sequenced as described above.

### *In vitro* editing analysis by poisoned primer extension

Total hepatic RNA was isolated from *Apobec-1*^-*/*-^ mice and treated with DNA-free reagent (Ambion). Resulting RNA (1 μg) was incubated for 3 h at 30°C with variable amount of hepatic S100 extract prepared from either WT or *Apobec-1*^-*/*-^ mice liver [9]. Following incubation with S100 extracts, the RNA was phenol/chloroform extracted, precipitated and resuspended in cDNA synthesis reaction mix (High Capacity cDNA Reverse Transcription kit (Applied Biosystems). Single-stranded DNA was then subjected to PCR amplification using primers specific for a Sanger-validated Apobec-1-dependent RNA target followed by poisoned primer extension using γ-ATP 5′ end-labeled primer annealing approximately three to six nucleotides downstream of the identified editing site as previously described [9]. Extension products were separated by electrophoresis on a 7 M urea-acrylamide gel and analyzed by autoradiography.

### Proteomics analysis

Total proteins were isolated from three WT and three *Apobec-1*^*-/-*^ intestine samples using a buffer containing 2% SDS, 30 mM Tris pH 8, supplemented with protease inhibitors (Complete EDTA-free, Roche), phosphatase inhibitors (PhosStop, Roche) and benzonase (25 U/μl, Sigma). Proteins were methanol/chloroform precipitated and resuspended in urea/thiourea buffer (6 M/2 M, 30 mM Tris, pH 8). Protein concentration was estimated using Bradford. Unlabeled samples were mixed with a lys6-labeled SILAC standard (analogously extracted from intestine from lys6-labeled mice; Silantes GmbH, Munich, Germany) at a ratio of 1:1. Samples were in-solution digested [48] using Lys-C (Wako Richmond, VA, USA) only. Peptides (200 μg) were separated by in-solution isoelectric focusing (Offgel fractionator, Agilent) into 12 fractions over a pH range of 3 to 10. Fractionation was performed according to the manufacturer’s protocol with adaptations. Glycerol and ampholytes in the separation buffer were reduced to 0.3% (original, 6%) and 0.1% (1%), respectively. Peptides were focused for 20 kVhr and harvested including a well washed with 50 μl 50:49:1 methanol:MilliQ:TFA for 15 minutes. Fractionated peptides were dried down with a speedvac (Eppendorf Hauppauge, NY, USA) prior to desalting using C_18_ StageTips according to [49]. The fractions obtained were individually submitted to liquid chromatography (LC) coupled to mass spectrometry (MS) [48]. After trimming to avoid ampholyte interference with data analysis using RecalOffline (ThermoFisher Scientific Waltham, MA, USA), mass spectrometry data were analyzed using the MaxQuant suite of algorithms (version 1.3.0.5; Cox and Mann, 2008). The data were searched against the *Mus musculus* UniProtKB protein sequence database (as of 8 May 2013) consisting of 79,342 entries, including canonical and isoform sequences. Search parameters were set as follows. Lys-C was selected with a maximum of two missed cleavages. Precursor mass tolerance was set to 20 ppm for the first search and to 6 ppm for the main search. Oxidized methionines and amino-terminal protein acetylation were allowed as variable, carbamidomethylation as a fixed modification. The false discovery rate for peptide and protein identification was set to 1%. Minimum peptide length was set to 7 with no maximum. Peptide identification by chromatography alignment and ID transfer ('match between runs') was enabled and led to identification of 3,210 proteins. Differentially expressed genes were identified by *t*-test (significance cutoff of 0.1) in R, a language and environment for statistical computing and graphics [50].

### Apobec-1-dependent editing sites: analysis of flanking sequence features

Analysis of bases flanking the editing sites was performed by aligning 10 nucleotides surrounding the editing sites (5 nucleotides immediately upstream and 5 nucleotides immediately downstream). Frequency plots and logos were generated using the WebLogo application [51,52]. Identification of consensus mooring sequence was performed by aligning 100 nucleotides surrounding the editing sites and looking for the consensus mooring sequence previously identified [16]. To determine the AU content of the targeted 3′ UTRs, the average AU content of both the full length 3′ UTR and a 101-bp window surrounding each editing site were compared to the distribution of 100,000 random sets of 101-bp windows in 3′ UTRs and whole 3′ UTRs of equivalent size.

### Gene expression analysis

Differential gene expression analysis was performed using the Tuxedo suite of tools [53]. RNA-seq reads were mapped onto the transcriptome (mm9 UCSC knownGene) using Bowtie version 2.0.0b7 [54] and TopHat version 2.0.5 [54]. Differentially expressed genes were called using fragments per kilobase of exon per million fragments mapped (FPKM) and reported as a measure of relative transcript abundance using Cufflinks version 2.0.2 [55].

### Statistical analysis

Degree of enrichment of the Apobec-1 targets was represented by the difference in hypergeometric distribution using one-tailed Fisher’s exact test. Correlation between editing frequency and fold protein expression is reflected by Spearman’s rho (ρ) rank correlation coefficient. Statistical significance was set at a *P* value <0.05. All analyses were performed using Graphpad Prism 4.0 (GraphPad Software, Inc. La Jolla, CA, USA).

## Abbreviations

ACF: Apobec-1 complementation factor; ADAR: adenosine deaminase acting on RNA; ApoB: apolipoprotein B; FPKM: fragments per kilobase of exon per million; miRNA: microRNA; PCR: polymerase chain reaction; SNP: single-nucleotide polymorphism; UTR: untranslated region; WT: wild type.

## Competing interests

The authors declare that they have no competing interests.

## Authors’ contributions

VB, NOD, JHN and AM designed the experiments. VB, EP, SS, MM, YL, SK, AMB, HBH and JG conducted the experiments. EP and AM undertook the bioinformatics analysis. VB, EP, NOD, JHN, SS, MM and AM analyzed the results. SS, AMB, HBH and JG undertook the proteomic analyses. VB undertook the RNA editing assays and polysome isolation and analyses. VB, NOD, JHN, SS and AM wrote the manuscript with input from all the authors. All authors read and approved the final manuscript.

## Authors’ information

Ali Mortazavi, Joseph H Nadeau and Nicholas O Davidson shared supervision of this work.

## Supplementary Material

Additional file 1 **The following supplemental data are available with the online version of this paper. Table S1A.** lists WT intestinal Apobec-1 exonic targets. **Table S1B**-**D.** list WT intestine discordant RNA targets. **Table S1E.** lists the WT liver Apobec-1 exonic targets. **Table S2.** shows alignment of mooring sequence-like motifs of WT intestine Apobec-1 RNA targets. **Table S3A.** lists intestinal RNA targets with increased editing efficiency correlating with increased Apobec-1 expression. **Table S3B.** lists hepatic C-to-U RNA editing targets shared in WT and *Apobec-1*^-*/*-^ mice following ad-Apobec-1 rescue. **Table S3C.** lists C-to-U editing targets shared between WT and *Apobec-1*^-*/*-^ + ad-Apobec-1 showing hyperediting following ad-Apobec-1 rescue. **Table S4.** shows alignment of mooring sequence-like motifs of WT liver Apobec-1 RNA targets. **Tables S5 and S6.** show, respectively, RNA expression of intestinal and hepatic Apobec-1 targets. **Tables S7 and S8.** show, respectively, intestinal and hepatic Apobec-1 editing sites in miRNA seed sequences. **Table S9.** lists the proteins differentially expressed between WT and *Apobec-1*^-*/*-^ intestine. **Table S10.** shows intestinal Apobec-1 RNA editing targets with altered protein expression. **Table S11.** lists miRNA seed sequences in Apobec-1 C-to-U RNA editing targets with altered RNA and protein expression. **Tables S12 and S13.** list primer sequences for PCR amplification of, respectively, intestine and hepatic 3' UTR Apobec-1 RNA targets. **Figure S1.** shows frequency plot analysis of nearest nucleotides flanking Apobec-1 3' UTR RNA editing sites. **Figure S2A,B.** shows that the extent of differential mRNA expression for each edited transcript is unrelated to the percent C-to-U editing. Click here for file
