# Supplementary material for: Genome-wide identification and functional analysis of Apobec-1-mediated C-to-U RNA editing in mouse small intestine and liver
Source: Genome Biol. 2014 Jun 19;15(6):R79. doi: 10.1186/gb-2014-15-6-r79 (PMC4197816; doi:10.1186/gb-2014-15-6-r79)
Supplement: Additional file 1 — The following supplemental data are available with the online version of this paper. Table S1A. lists WT intestinal Apobec-1 exonic targets. Table S1B-D. list WT intestine discordant RNA targets. Table S1E. lists the WT liver Apobec-1 exonic targets. Table S2. shows alignment of mooring sequence-like motifs of WT intestine Apobec-1 RNA targets. Table S3A. lists intestinal RNA targets with increased editing efficiency correlating with increased Apobec-1 expression. Table S3B. lists hepatic C-to-U RNA editing targets shared in WT and Apobec-1-/- mice following ad-Apobec-1 rescue. Table S3C. lists C-to-U editing targets shared between WT and Apobec-1-/- + ad-Apobec-1 showing hyperediting following ad-Apobec-1 rescue. Table S4. shows alignment of mooring sequence-like motifs of WT liver Apobec-1 RNA targets. Tables S5 and S6. show, respectively, RNA expression of intestinal and hepatic Apobec-1 targets. Tables S7 and S8. show, respectively, intestinal and hepatic Apobec-1 editing sites in miRNA seed sequences. Table S9. lists the proteins differentially expressed between WT and Apobec-1-/- intestine. Table S10. shows intestinal Apobec-1 RNA editing targets with altered protein expression. Table S11. lists miRNA seed sequences in Apobec-1 C-to-U RNA editing targets with altered RNA and protein expression. Tables S12 and S13. list primer sequences for PCR amplification of, respectively, intestine and hepatic 3' UTR Apobec-1 RNA targets. Figure S1. shows frequency plot analysis of nearest nucleotides flanking Apobec-1 3' UTR RNA editing sites. Figure S2A,B. shows that the extent of differential mRNA expression for each edited transcript is unrelated to the percent C-to-U editing. [file gb-2014-15-6-r79-S1.pdf]

Supplemental Table 1A. Wild-Type intestinal Apobec-1-dependent exonic targets

RNA	Chr	Position	Ref Base	RNA-seq	Sanger	AA (C-U)
1. ApoB	12	8014860 (+)	C	98%	100% (20/20)	Glu-Stop
ApoB	12	8015181 (+)	C	14%	30% (6/20)	His-Tyr
2. Cir1	2	73122939 (-)	G	22%	0 (0/19)	Ser-Phe
3. Gak	5	109021367 (-)	G	20%	31% (6/19)	Thr-Leu
4. Sgpl1	10	60565907 (-)	G	12%	46% (6/13)	Thr-Leu
5. Tonsl	15	76463630 (-)	G	12%	0 (0/20)	Pro-Leu
6. Abi1	2	22797404 (-)	G	10%	0 (0/21)	His-His

Supplemental Table 1B. WT intestine Sanger discordant RNAs

	RNA	Chr	Position	RNA-seq	Reads	Sanger	(Edited/Total)
1.	Mdh1	11	21456926 (-)	79%	14	0%*	(0/18)
	Mdh1	11	21456925 (-)	67%	15	0%*	(0/18)
2.	Cflar	1	58789789 (+)	67%	18	0%†	(0/20)
3.	Apoo	X	91662208 (+)	50%	134	0%†	(0/20)
4.	Dpp4	2	62169419 (-)	50%	78	0%*	(0/22)
5.	Tspan6	X	130426116 (-)	45%	39	0%*	(0/20)
	Tspan6	X	130425814 (-)	33%	12	0%*	(0/21)
6.	Smek1	12	102277863 (-)	42%	49	0%*	(0/22)
7.	Asap2, Itga1bp1	12	21275864 (+)	39%	116	0%§	(0/20)
8.	Api5	2	94253833 (-)	38%	107	0%*	(0/22)
9.	Hpgd	8	58799127 (+)	36%	370	0%‡	(0/20)
10.	Ugt1a1	1	90115552 (+)	34%	217	0%*	(0/20)
11.	Kctd12	14	103379508 (-)	32%	25	0%*	(0/18)
12.	Asph	4	9503468 (-)	30%	40	0%*	(0/21)

† Island-mapping artefact
§ Overlapping antisense 3'UTRs
‡ Homopolymer
* No obvious reason for not being detected by Sanger sequencing
(+) Sense strand
(-) Antisense strand

Supplemental Table 1C. RNA-seq discordant cohort targets

RNA	Chr	Position	Rosenberg RNA-seq	RNA-seq	Reads	Sanger	(Edited/Total)
1. BC003331	1	152208563 (-)	74%	BT§	30	18%	(4/22)
2. Ptpn3	4	57203753 (-)	67%	BT§	24	45%	(8/18)
3. Rb1	14	73595382 (-)	33%	BT§	51	18%	(4/22)
4. Abcb7	X	101478733 (-)	nd	BT‡	9	68%	(15/22)

BT below threshold
§ Does not have 1 read per strand
‡ Less than 10 read coverage
(-) Antisense strand

Supplemental Table 1D. Sanger discordant cohort targets in isolated enterocytes (IE)

					WT				*Apobec-1* $^{Int/O}$			
	**RNA**	**Chr**	**Position**	**Rosenberg RNA-seq**	**RNA-seq**	**Reads**	**Sanger**		**RNA-seq**	**Reads**	**Sanger**	
							Mucosa (Edited/Total)	IE (Edited/Total)			Mucosa (Edited/Total)	IE (Edited/Total)
1.	Casp6	3	129616676 (+)	50%	55%	465	0% (0/20)	36% (8/22)	16%	611	5% (0/18)	0% (0/22)
2.	Dpyd	3	119135667 (+)	32%	40%	55	0% (0/20)	0% (0/22)	BT*†§	15	0% (0/17)	0% (0/22)
	Dpyd	3	119135669 (+)	28%	14%	59	0% (0/20)	0% (0/22)	BT*†§	18	0% (0/17)	0% (0/22)
3.	Iqgap2	13	96397211 (-)	38%	32%	220	0% (0/22)	0% (0/22)	95%	113	0% (0/22)	0% (0/22)
	Iqgap2	13	96397289 (-)	23%	30%	1042	0% (0/22)	0% (0/22)	BT†	447	0% (0/22)	0% (0/22)
4.	Atf2	2	73654730 (-)	29%	17%	161	0% (0/20)	18% (4/22)	68%	79	0% (0/22)	26% (6/23)

BT Below Threshold
IE Isolated enterocytes
* Less than 3 reads supporting C-to-U editing
† Less than 10% C-to-U editing
§ Does not have 1 read per strand
(+) Sense strand
(-) Antisense strand

Supplemental Table 1E. Wild-type liver Apobec-1-dependent exonic targets

RNA	Chr	Position	Ref Base	RNA-seq	Sanger	AA (C-U)
1. ApoB	12	8014860 (+)	C	72%	70% (7/10)	Glu-Stop
2. BC005561	5	104949524 (+)	C	50%	9% (2/22)	Leu-Leu
3. Klc1	12	113027553 (+)	C	22%	(0/18)	His-His
4. Cinp	12	112112206 (-)	G	14%	(0/18)	Pro-Pro
5. Rit1	3	88533081 (+)	C	13%	(0/20)	Pro-Pro
6. Sf3b1	1	55062747 (-)	G	10%	(0/21)	Pro-Ser

Supplemental Table 2. Alignment of mooring sequence-like motifs of WT intestine Apobec-1 RNA targets

RNA	Chr	Position	WRAUYANUAU	RNA-seq
Apob	12	8014860	5′-AAAACUCUCUCAACUUGAGACAUACGCGAUACAAUUUGAUCAGUAUAUUAAAGAUAAUUAUGAAU-3′	98%
Cd36	5	17288955	5′-UAUAUAUAUAUGCAAUAAGUCACAGCAUAUUUCAAAAGAUUAAUAUGUCACUAUAGGCAAUAUUU-3′	84%
Reps2	X	158851906	5′-AUACUUUUCUCCAGUAUAUUGUUGGUCACUCACAAAUGAUUCAUUUGAACACAUUUUAGGGAUAA-3′	75%
Siglec5	7	50614573	5′-CUAAAGUUAUUCUGUUUUGAAGUGUGACUGUGUUGUAGCUACAUAUAUCUCAAAUUGAACUCAUA-3′	72%
Fmn1	2	113556683	5′-UAUGCGUGUACAUAUAUUUUCCUUCUUGACACUACUUGAUCAUUAUAUCAAGUGUAUUUUUGUAC-3′	71%
0610010O12Rik	18	36562329	5′-GCCACCUCCGAUAAGUGUGCCUGGCCCCAUCUCUUCUGAUUGCUAUAAAGUGGCUAGCUCUGCGC-3′	67%
Mcmbp	7	135841366	5′-AAGUCAUUUGAAAGAAAAAAAGAACAAAACACACUGUGAUCACUAUUUUGUUUUCAUAAAGGACA-3′	63%
Man2a1	17	65104330	5′-AAUCCUUUGUCUAGAAGAAAAUGUUUACUUUCAUUGUGAUUAUUUUGCUCAUUGAAAUCAACAGG-3′	60%
Herc2	7	63486942	5′-ACUCUGAAUUUGUUUAUUAAAAGCUUUUUGAACAUUAAAUUAUUAUUUCUUCAAUGCAUUUAGAA-3′	60%
Ddx60	8	64516163	5′-UUCUAAAUUAUUGAGGAUUGAAGGAAAAAAAAUACAAGAAUAUUACAUAUAUACAUUUAAAGACA-3′	59%
Tmem195	12	38308269	5′-UCUUUACCUAUGCUGUUCACAUUUGUUUUUCUGUUGAAAUUACUAUGAAUUAUUAUUUUUAUUUU-3′	56%
Mtmr2	9	13610423	5′-UUUGUGAUAUUUAAUAUAGUAAAAAGCAUUGUCUUGAGAUUAUUAUGGCAUAGUAGGUUUUUUUU-3′	53%
Cyp2c65	19	39168358	5′-UUCAGCCCUGUUCUUAUCUGAUGGUGAACUACUAACAUAUUAUUAUCUUAAAACUGAGAAAUAUG-3′	50%
Cnih	14	47395982	5′-UCUACUUGGAUAAAGAAUUUAAAUGGAAUUACGUAUAAAUUAAUAUAAAAUGAUUACCUCUGGUG-3′	48%
Atp11c	X	57477477	5′-UUAAUUUAAUUUGUUCUCAUCUAUCAGUAUUACUUUUGAGUAUUUUGUUAGGUGUCAAUCACAGC-3′	46%
Sh3bgrl	X	106356686	5′-CUUGAUUUUUAUAAAGAAACACACAUCUUUUACUUCAAAUUACUAUUAUCAUCAUACCUGCUCUU-3′	45%
Fgl2	5	20883372	5′-AUCUUAUUUCUUUUUGCUUUUUAAAAAAGACUAAGAAGAUUUUGAUAUUAUACAAUUCAUGUGUU-3′	42%
Nrld2	14	19036726	5′-UAAUUGCAGAUAAAAAAAAUUUGUUGGGUGUUUCAGUAGAUUAAUAUAUAGAUUUCAUAUCUACAU-3′	39%
Tmem135	7	96290044	5′-AUCUAUGUAGAAAUAUAAUAAGAAGAGUAUACUUAAAAAUUACUUUAAAAGAUAUCUUUAGUUCA-3′	39%
Slc4a4	5	89668527	5′-UAUAUUACUAAUAAUGUUUUUCCAAUAUGAAACCUAAAAUUCCUAUAACAUAGUAUUUUACAGUU-3′	38%
Dpyd	3	119134696	5′-UUCUUUCCAAAUUUUAUAGCUGAACAAUAACAAUUGUAAUCAUUAUGAUCAGUUCUUUCCAAAUU-3′	38%
Ttc9c	19	8885447	5′-AAUUUCUUAUACUCCUUUAAUAUUUCAGAGACAUUCAGAUUAUUUUCCUCCAUAAAAGUAUCUUC-3′	37%
Yes1	5	32989151	5′-CCUUAUAAUACAAAUUGUAUAGUAAGGAUACAACACUAAUUAAUAUGUUUUCAGUUUAAUUUGUU-3′	36%
1110020G09Rik	15U	9038469	5′-CUUCCUAACUUUGUGGAAUUGGCCAUGUGUAUCAGUUGAUUAGUACAUUAUUUUUAUAGUACUUA-3′	36%
Actr2	11	19963383	5′-UGAAACAAGAGAAGCUGUAUCACAGAAAAUACAAAAUGAUGAUUCUGAGAGAAAUAUUUUCCUCA-3′	35%
Kctd12	14	103379573	5′-GUCUAUCGAGCUAUUUUGAGUCCUAAACUUAGUUGCUAAUACUUGUAUAACCUAACCAAAGAGUU-3′	35%

Nr3c1	18	39571801	5′-UGGAUGUGAAAUUUAUGGUGCCUAAGAAAUA**C**CACU**UGA**AG**AUUAU**CAAUGACAGUGUUAAGUUU-3′	33%
Skil	3	31018375	5′-UUUUUCUUUUAAAUUAUAAAAUACUAAAUAU**C**UUGA**AGAUUAUU**U**U**GGACUUUUGUAUGUUUAAA-3′	33%
Ccny	18	9315769	5′-AUCCCUCUCCUGCCUAGUUCCAAGAACAUU**C**UAUCU**AGAUUAAUAU**GGGUGCUUGUGCCUUCCAG-3′	32%
Rab1	11	20125336	5′-UCCUGGUAGAGUUAUAAAUGGAAAGAUUACA**C**UAUC**UGAUUAAUA**GUUUCUUCAUACUCUGCAUA-3′	32%
mCG_2776	6	8378189	5′-CAUUUCCUUGCCGUGAAAAUUUUAAGUCAUUU**C**UUU**UGAUUAGU**UAUAAUUUAUUAAUUCUGUGC-3′	31%
Lrba	3	86586529	5′-GUUAUUUUGGUGUCAUAUAAUUCUACUUUUU**C**UAGU**AGAUU**G**CU**U**U**AUGGAAUUCUGUGAAAAUA-3′	31%
Dek	13	47181166	5′-GAACUAUUGUGAAUUUUAGUGAA**C**UUAAUGUUAGCU**UG**C**UUAA**G**A**CAUUAGUUUUAAUCAAAGCU-3′	31%

ApoB RNA sequences surrounding the canonical editing site C6666 is shown as reference in relation to mooring sequence content and position with editing site. Consensus mooring sequence is shown in dark blue: W (A/U), Y (pyrimidines), R (purines).
Edited cytidines are indicated in red bold character. Matched nucleotides within the consensus motif are indicated in dark blue. Light orange indicates mismatches.

Supplemental Table 3A. A subset of C-to-U RNA editing targets in intestine from indicated genotypes show increased editing efficiency with increased Apobec-1 expression

			WT				*Apobec -1* $^{Int/+}$				*Apobec -1* $^{Int/OLo}$				*Apobec -1* $^{Int/OHi}$			
**RNA**	**Chr**	**Position**	**RNA -seq**	**Reads**	**Sanger**	**(Edited /Total)**	**RNA-seq**	**Reads**	**Sanger**	**(Edited /Total)**	**RNA-seq**	**Reads**	**Sanger**	**(Edited /Total)**	**RNA-seq**	**Reads**	**Sanger**	**(Edited /Total)**
**1. Cmtm6**	9	114658289 (+)	54%	305	75%	(15/20)	56%	152	36%	(8/22)	21%	249	**14%**	(3/21)	59%	213	**40%**	(8/20)
	9	114658301(+)	12%	194	20%	(4/20)	13%	112	9%	(2/22)	BT†	247	**0%**	(0/22)	12%	149	**10%**	(2/20)
**2. Usp25**	16	77116537 (+)	68%	44	**58%**	(11/19)	75%	49	**68%**	(13/19)	71%	67	50%	(10/20)	87%	31	39%	(7/18)
**3. Actr2**	11	19963383 (-)	35%	52	**41%**	(9/22)	62%	50	**57%**	(16/28)	48%	82	**0%**	(0/21)	74%	35	**85%**	(17/20)
**4.Atp6ap2**	X	12193607 (+)	30%	162	**28%**	(5/18)	57%	96	**62%**	(18/21)	52%	75	**4.5%**	(1/22)	79%	104	**53%**	(10/19)
**5. Yme1l1**	2	23052720(+)	22%	116	**21%**	(4/19)	45%	66	**31%**	(4/13)	19%	118	57%	(12/21)	53%	55	59%	(13/22)
**6. Rab1**	11	20125336(+)	32%	508	**15%**	(3/20)	53%	248	**64%**	(14/22)	43%	392	60%	(12/20)	75%	345	45%	(10/22)

BT Below Threshold
† Less than 10% C-to-U editing
(+) Sense strand
(-) Antisense strand
Bold numbers indicate editing efficiency increased with increasing Apobec-1 expression

Supplemental Table 3B. Hepatic C-to-U RNA editing targets shared in wild-type and *Apobec-1*$^{-/-}$ mice following ad-Apobec-1 rescue

Genotype:			WT				*Apobec-1*$^{-/-}$ + Ad-Apobec-1			
RNA	Chr	Position	RNA-seq	Reads	Sanger	(Edited/Total)	RNA-seq	Reads	Sanger	(Edited/Total)
1. Serinc1	10	57235791 (-)	38%	122	9%	(2/21)	77%	122	77%	(17/22)
2. Aldh6a1	12	85772761 (-)	12%	291	14%	(3/22)	51%	291	50%	(11/22)
3. Tmem30a	9	79617629 (-)	11%	71	8%	(2/23)	37%	71	21%	(4/19)
4. Sh3bgrl	X	106355759(+)	BT†§	26	17%	(3/18)	49%	11	65%	(13/20)
5. Cyp4v3	8	46391931 (-)	BT†	350	14%	(3/22)	65%	515	67%	(14/21)
6. Casp6	3	129616676 (+)	BT*†§	36	0	(0/22)	56%	25	35%	(7/20)
7. Rrbp1	2	143811725 (-)	BT†§	349	0	(0/22)	54%	234	50%	(10/20)
8. Hprt	X	50374459 (+)	BT*†§	86	0	(0/22)	32%	132	35%	(7/20)

BT Below Threshold
*Does not have 3 reads demonstrating C-to-U editing
†Does not have at least 10% C-to-U editing
§ Does not have 1 read per strand
(+) Sense strand
(-) Antisense strand

Supplemental Table 3C. **C-to-U RNA editing targets shared between wild-type and *Apobec-1*$^{-/-}$ + ad-Apobec-1 show hyperediting following ad-Apobec-1 rescue**

RNA	Chr	Position	Sanger	(Edited/Total)
1. Aldh6a1	12	85772583 (-)	9 %	(2/22)
		85772599 (-)	27%	(6/22)
		85772780 (-)	9%	(2/22)
		85772782 (-)	18%	(4/22)
		85772795 (-)	9%	(2/22)
		85772796 (-)	18%	(4/22)
		85772799 (-)	14%	(3/22)
		85772963 (-)	14%	(3/22)
		85772966 (-)	18%	(4/21)
2. Tmem30a	9	79617471 (-)	5%	(1/19)
		79617478 (-)	5%	(1/19)
		79617484 (-)	5%	(1/19)
		79617487 (-)	5%	(1/19)
		79617542 (-)	5%	(1/19)
		79617729 (-)	5%	(1/19)
3. Sh3bgrl	X	106355693 (+)	20%	(4/20)
		106355707 (+)	20%	(4/20)
		106355745 (+)	15%	(3/20)
		106356675 (+)	25%	(5/20)
		106356677 (+)	10%	(2/20)
		106356686 (+)	45%	(9/20)
		106356731 (+)	35%	(7/20)
		106357058 (+)	10%	(2/20)
4. Cyp4v3	8	46391814 (-)	28%	(6/21)
		46391816 (-)	33%	(7/21)
		46391894 (-)	33%	(7/21)
		46391896 (-)	5%	(1/21)
		46391937 (-)	9%	(2/21)
		46391939 (-)	38%	(8/21)
		46392029 (-)	19%	(4/21)
		46392203 (-)	19%	(4/21)
5. Casp6	3	129616581 (+)	5%	(1/20)
		129616703 (+)	15%	(3/20)
		129616728 (+)	10%	(2/20)
6. Rrbp1	2	143811727 (-)	15%	(3/20)
7. Hprt	X	50374397 (+)	5%	(1/20)
		50374403 (+)	5%	(1/20)
		50374419 (+)	5%	(1/20)
		50374440 (+)	5%	(1/20)

Supplemental Table 4. Alignment of mooring sequence-like motifs of WT hepatic Apobec-1 RNA targets

RNA	Chr	Position	WRAUYANUAU	RNA-seq
ApoB	12	8014860	5′-AAAACUCUCUCAACUUGAGACAUACGCGAUACAAUUUGAUCAGUAUAUUAAAGAUAAUUAUGAAU-3′	72%
Serinc1	10	57235791	5'-UUACUGCCAUGCAAUUGAUAUAGUAAAUCGACUAGAAAAUUAGUAUGCCACUUAAUUAAGAUAAA-3'	38%
Dcn	10	96980667	5'-CAUAAAGUCAAAUGCCGCCGAACUCUAGCAAUGUAUUAAUCUCCUUUAUUAUUGGUGAAGCCUUA-3'	30%
Cd36	5	17288955	5′-UAUAUAUAUAUGCAAUAAGUCACAGCAUAUUUCAAAAGAUUAAUAUGUCACUAUAGGCAAUAUUU-3′	24%
Cybb	X	9012717	5'-GUUUAUGGAAUUUGAAUACUUGCAGCCAUUGUAUAUGAACAGUCUGGUGAGUGGGAGAAGAUGAU-3'	23%
Cybb	X	9012852	5'-GCUGCCAGCUGCCAGCACAGCUCUUUCUGUACUUAGUUCUUAGAAUUUAGUAACAGAUACUGAGA-3'	23%
Colec10	15	54297696	5'-GCUGCCUUUUAGAAUAUCAGGCCUUCAAACUACAGCUGAAUCCUUUCAUGUAUGAGGGAAAAUAU-3'	18%
Ube2l3	16	17152203	5'-AAUCCACAUCCCCCUGGUUCCUUGAAGUGCCCGGGUCUCGUCCUGCUCUUCCCACAAUGGCAGAG-3'	16%
Dcn	10	96980535	5'-GUCUGUAGUUGUGAACUGAGUUAUCAAAGUCUGAUGUAAUCAUAAAUGUCAACCACUUAGUAAAG-3'	14%
Cybb	X	9013390	5'-AUUAAUGAGUCAAUUUUAACUAUUUGUGGCAAAGUUUGAUCUCAGUUACAAGGACUCUGAAAACA-3'	14%
Abcc9	6	142538042	5'-CUAGAGUUUAAUUCUUUAUCAGCCAGGCAAUGACUCUGAACUCUGAAAUGCUAUAUUAUUUAGGA-3'	14%
Colec10	15	54295026	5'-UUAUGACUUUGCCCAUGACUAACUGGUGGUGAGAAGAGAUUACUGGCACUGACUAUGUGAAUUCA-3'	13%
Aldh6a1	12	85772761	5'-GAAAUCAAUACUCCAGCCAUUAAUGCAGAUACAAAUAGAUCCUUAGAUAAAAUCUUUCCACAAAU-3'	12%
Abcc9	6	142538035	5'-UUCUUUAUCAGCCAGGCAAUGACUCUGAACUCUGAAAUGCUAUAUUAUUUAGGACCCAAAAGAAA-3'	11%
Tmem30a	9	76917629	5'-AUUUUAAAUCAAUCGGUCUGGGUAACAUAUCAGUUUAGAUUAAUAUGUGCUUAAAAGAAGAAAAA-3'	11%
Mpeg1	19	12539179	5'-GUCUUAGAAAUUAUGUAUCUAUUCACCACAACACAUCAGUGAGCUAUCUUCUACGAAAGAUGAUG-3'	11%
Usp25	16	77116537	5'-CGUUUGUUCUUUUCUGUAUGUAUAAAUAAUACAAGGUGAUUAUUUCAAGAGAAACCUGAAAAAGU-3'	BT
Sh3bgrl	X	106355759	5'-UGCCAAGGCAUUUCUGUUCUGUAAGGAUUUUACAAAAAAUCAAUAUGCUCCUUCUUUUAAAUUUC-3'	BT
Cmtm6	9	114658289	5'-UAAGAAGGGAAAUAUACUUAUUGCACAAGACUUUUAUAAUUACUAUACUUAAAUUAUGCUCUAUG-3'	BT
Sep15	3	144259976	5'-AUUAUCACAGCACCUAGACAAUAACUUAGUUUUGCAUGCUUACAUUGGUCAUCCUUUUUAUGUAC-3'	BT
Cyp4v3	8	46391931	5'-AAUCUCAGAAUUAAGAAAAUGUUCUCAUUGACUUGGAAAUUCAUAUAUCAUUUGAAAAUGUUGGG-3'	BT
Rnf128	X	136207009	5'-AGCAUAGUUUGUGAGAAAAAUGUGAACUGAAUUGGUUGAUCAGUAUAUUAGUGACACCACACAAG-3'	BT
B2m	2	121978638	5'-ACAAUAGUUGAUCAUAUGCCAAACCCUCUGUACUUCUCAUUACUUGGAUGCACUUACUCAUCUUU-3'	BT

ApoB RNA sequences surrounding the canonical editing site 6666 is shown as reference in relation to mooring sequence content and position with targeted cytidine. Consensus mooring sequence is shown in blue: W (A/U), Y (pyrimidines), R (purines). Edited cytidines are indicated in bold red. Nucleotides matching the consensus motif are indicated in dark blue. Light orange nucleotides indicate mismatches. BT: Below Threshold.

Supplemental Table 5. RNA expression of intestinal Apobec-1 targets

	FPKM			Q-PCR		
**RNA**	**WT**	***Apobec-1*** $^{-/-}$	**Fold change**	**WT**	***Apobec-1*** $^{-/-}$	**Fold change**
1. **Siglec5***	9	1	9	1± 0.3	0.1 ± 0.06§	10
2. **Kctd12***	16	3	5.4	1 ± 0.2	0.4 ± 0.1 §	2.5
3. **Cyp2c65***	109	45	2.4	1 ± 0.2	0.3 ± 0.1 §	3
4. **Bche**	14	6	2.3	1 ± 0.2	0.8 ± 0.4	1.2
5. **Cd36***	21	9	2.3	1 ± 0.1	0.6 ± 0.3†	1.7
6. **Mfsd7b***	39	18	2.2	1 ± 0.2	0.4 ± 0.02 †	2.5
7. **Aldh6a1***	4	2	2	1 ± 0.06	0.3 ± 0.05§	3
8. **Ank3**	12	6	2	1 ± 0.3	2 ± 0.4	0.5
9. **Tmem195**	24	13	1.8	1 ± 0.1	0.8 ± 0.5	1.2
10. Fmn1	5	3	1.7			
11. **Ptpn3***	12	7	1.7	1 ± 0.03	0.4 ± 0.07§	2.5
12. **Rb1**	7	4	1.7	1 ± 0.06	0.9 ± 0.08	1
13. Herc2	10	6	1.6			
14. Gramd1c	13	8	1.6			
15. **Cyp4v3***	288	182	1.6	1 ± 0.1	0.7 ± 0.03	1.4
16. Lrrc19	8	5	1.6			
17. **Slc4a4**	26	17	1.5	1 ± 0.08	1 ± 0.05	1
18. Man2a1	12	8	1.5			
19. **App**	142	93	1.5	1 ± 0.1	1 ± 0.25	1
20. **Fgl2**	24	17	1.4	1 ± 0.1	0.9 ± 0.1	1
21. **1110020G09Rik**	11	8	1.4	1 ± 0.2	0.8 ± 0.1	1.2
22. 2010106E10Rik	162	114	1.4			
23. **Serinc1***	17	12	1.4	1 ± 0.1	0.9 ± 0.02	1
24. Ccny	11	8	1.4			
25. Dpyd	37	27	1.4			
26. **Rab1**	150	113	1.3	1 ± 0.06	1.3 ± 0.08§	0.8
27. Tmem135	23	17	1.3			
28. Nr1d2	10	8	1.2			
29. Mtmr2	6	5	1.2			
30. **Cmtm6***	50	41	1.2	1 ± 0.2	1.2 ± 0.2	0.8
31. Reps2	2	2	1			
32. Lrba	14	12	1			
33. Skil	13	11	1			
34. **Clic5**	56	48	1	1 ± 0.08	1.4 ± 0.1	0.7
35. **Mcmbp***	7	7	1	1 ± 0.23	1.2 ± 0.3	0.8
36. 0610010O12Rik	297	266	1			
37. mCG_2776	6	6	1			
38. Ttc9c	5	5	1			
39. **Sult1d1***	65	59	1	1 ± 0.12	1.3 ± 0.03	0.8
40. Abcb7	6	6	1			
41. Cnih	42	39	1			
42. **Sep15***	59	56	1	1 ± 0.07	1 ± 0.05	1
43. **Usp25***	15	15	1	1 ± 0.1	1.4 ± 0.08	0.7
44. Tmbim6	1063	1018	1			

45. Nr3c1	12	12	1			
46. **BC013529***	22	22	1	1 ± 0.03	1 ± 0.1	1
47. Atp11c	1	1	1			
48. Yes1	7	7	1			
49. **Rnf128**	128	131	1	1 ± 0.3	0.7 ± 0.05	1.4
50. **Sh3bgrl***	12	12	1	1 ± 0.2	1.3 ± 0.1	0.8
51. Hprt	24	25	1			
52. B2m	1826	1911	0.9			
53. **Actr2***	47	50	0.9	1 ± 0.1	1.4 ± 0.04†	0.7
54. **Rrbp1***	167	187	0.9	1 ± 0.04	1 ± 0.05	1
55. **Tmem30a***	17	19	0.9	1 ± 0.08	1 ± 0.2	1
56. **Ddx60***	4	5	0.8	1 ± 0.4	1.6 ± 0.3	0.6
57. **BC003331***	4	6	0.7	1 ± 0.06	1.4 ± 0.2	0.7
58. Dek	5	7	0.7			

FPKM and Q-PCR fold changes are indicated as wild-type/*Apobec-1*$^{-/-}$ ratio. RNAs indicated in blue showed decreased expression (FPKM) in *Apobec-1* $^{-/-}$ mice. RNA indicated in red were up-regulated in *Apobec-1* $^{-/-}$ mice. RNAs indicated in black showed no change in expression between wild-type and *Apobec-1* $^{-/-}$ animals. RNA indicated in bold were analyzed by quantitative PCR. * indicates RNAs whose differential expression between *Apobec-1*$^{-/-}$ and WT was confirmed by quantitative PCR. Data are expressed as mean ± SDE (n= 5 animals/genotype). § P value <0.01; † P value <0.05. 47% (27/57) of Apobec-1 RNA targets showed decreased FPKM levels in absence of Apobec-1.

Supplemental Table 6. RNA expression of hepatic Apobec-1 targets

	FPKM		Q-PCR	
**RNA**	**WT**	***Apobec-1*** $^{-/-}$	**WT**	***Apobec-1*** $^{-/-}$
1. **Cd36** *	24	13	1 ± 0.1	0.4 ± 0.05§
2. Aldh6a1	119	111	1 ± 0.1	0.3 ± 0.06§
3. Usp25	7	9	1 ± 0.1	0.6 ± 0.05†
4. Cybb	4	7		
5. Colec10	6	9		
6. **Rnf128**	36	31	1 ± 0.3	1.6 ± 0.3
7 Sep15	104	97		
8. **Sh3bgrl**	24	27	1 ± 0.4	1 ± 0.03
9. Mpeg1	17	21		
10. Cmtm6	28	34		
11. **Tmem30a**	42	51	1 ± 0.2	1 ± 0.1
12. Abcc9	4	5		
13. B2m	2539	2799		
14. **Dcn**	68	70	1 ± 0.1	0.8 ± 0.1
15. Cyp4v3	143	145		
16. Ube2l3	41	41		

RNAs indicated in blue showed by FPKM analysis and quantitative PCR (bolded blue) or by quantitative PCR only (light blue), decreased expression in *Apobec-1* $^{-/-}$ mice. RNAs indicated in red were shown by FPKM analysis to be up-regulated in *Apobec-1* $^{-/-}$ mice. RNAs indicated in black showed no change in expression between wild-type and *Apobec-1* $^{-/-}$ mice, either by FPKM analysis (light black) or by FPKM analysis and quantitative PCR (bolded black). Quantitative PCR data are expressed as mean ± SDE (n=4 animals/genotype). § P value < 0.01; † P value < 0.05. One third of the hepatic Apobec-1 RNA targets showed alteration of expression in absence of Apobec-1 (*i.e.* in absence of RNA editing).

Supplemental Table 7. Intestine Apobec-1 editing sites in miRNA seed sequences

Gene	Editing site	*Apobec-1*$^{-/-}$ miRNA seed match (C)	WT miRNA seed match (U)
1. **Tmem195***	Chr12: 38308269	mmu-miR-205-3p<br>mmu-miR-1912-3p	mmu-miR-1192
2. **Siglec5***	Chr7: 50614573	mmu-miR-128-3p<br>mmu-miR-5620-3p<br>mmu-miR-582-5p<br>mmu-miR-6539<br>mmu-miR-707	mmu-miR-154-3p<br>mmu-miR-96-3p<br>mmu-miR-539-3p<br>mmu-miR-374b-5p
3. **App**§	Chr16: 84954758		mmu-let-7f-2-3p<br>mmu-let-7f-1-3p
4. **Sep15**†	Chr3: 144259976	mmu-miR-6374<br>mmu-miR-6414	mmu-miR-126-5p
5. **Mcmbp**†	Chr7: 135841366	mmu-miR-466d-5p<br>mmu-miR-466k<br>mmu-miR-6358<br>mmu-miR-6376	mmu-miR-16-1-3p<br>mmu-miR-154-3p
6. Cnih†	Chr14: 47395982		mmu-miR-297a-3p<br>mmu-miR-297b-3p<br>mmu-miR-297c-3p<br>mmu-miR-467b-3p<br>mmu-miR-467e-3p<br>mmu-miR-467g
7. Dpyd	Chr 3: 119134696	mmu-miR-5128<br>mmu-miR-872-5p	mmu-miR-126-5p<br>mmu-miR-503-3p
8. 2010106E10Rik*	Chr X: 109671648	mmu-miR-6360	
9. B2m†	Chr2: 121978638		mmu-miR-26b-5p<br>mmu-miR-26a-5p
10. Reps2†	ChrX: 158851906	mmu-miR-3102-5p<br>mmu-miR-702-5p<br>mmu-miR-804	mmu-miR-467b-5p<br>mmu-miR-467h<br>mmu-miR-5617-5p<br>mmu-miR-6380<br>mmu-miR-668-5p
11.0610010O12Rik†	Chr18: 36562329	mmu-miR-143-3p	mmu-miR-5619-3p
12. Mtmr2†	Chr9: 13610423		mmu-miR-294-5p<br>mmu-miR-292-5p
13. mCG_2776†	Chr6: 8378189	mmu-miR-186-5p	
14. Sh3bgrl§	ChrX: 106356686		mmu-miR-203-3p<br>mmu-miR-32-3p

RNAs indicated in blue were shown to be down regulated in *Apobec-1*$^{-/-}$ mice by either FPKM analysis only (light blue) or FPKM and quantitative PCR (bolded blue). RNAs indicated in black showed no change of expression between wild-type and *Apobec-1*$^{-/-}$ mice by either FPKM analysis only (light black) or FPKM and quantitative PCR (bolded black). * RNAs where C to U RNA editing eliminates miRNA seed sequences, correlating with increased RNA level expression in wild-type mice (editing competent)

compared to *Apobec-1* $^{-/-}$ (Supplemental Table 5). § RNAs where C to U RNA editing creates miRNA seed sequences but no concomitant decreased RNA expression was observed in wild-type animals (Supplemental Table 5). † RNAs where C to U RNA editing either creates or eliminates miRNA seed sequence but no change in RNA expression (FPKM and/or quantitative PCR) was observed between wild-type and *Apobec-1* $^{-/-}$ mice (Supplemental Table 5).

Supplemental Table 8. Hepatic Apobec-1 editing sites in miRNA seed sequences

**Gene**	**Editing site**	***Apobec-1*$^{-/-}$ miRNA seed match (C)**	**WT miRNA seed match (U)**
1. **Cd36**§	Chr5: 17288955		mmu-miR-542-3p
2. Colec10*	Chr15: 54297696		mmu-miR-337-3p
3. Dcn	Chr10: 96980535		mmu-miR-3066-3p
4. Ube2l3	Chr16: 17152203	mmu-miR-1298-3p	
5. Mpeg1	Chr10: 12539179		mmu-miR-181b-2-3p<br>mmu-miR-468-5p

RNA indicated in bolded blue was shown by FPKM analysis and quantitative PCR to be down-regulated in *Apobec-1* $^{-/-}$ mice (Supplemental Table 6). RNA indicated in light red was shown by FPKM analysis to be up-regulated in *Apobec-1*$^{-/-}$ mice. * RNA where C –to-U RNA editing creates a miRNA seed sequence with concomitant decreased RNA expression in editing-competent wild-type mice. § RNA where C-to- U RNA editing creates a miRNA seed sequence but no concomitant decreased RNA expression was observed in editing-competent wild-type mice. Indicated in black are RNAs where C-to-U RNA editing either create or eliminate miRNA seed sequences without concomitant alteration of RNA expression between wild-type and *Apobec-1*$^{-/-}$ mice (Supplemental Table 6).

Supplemental Table 9. 893 proteins differentially expressed between WT and *Apobec-1 –/–*

Gene names	P Value (t-test)
0610010K14Rik;Bap18	0.020509134
1810020D17Rik	0.033168157
2210016F16Rik	0.053663752
2210407C18Rik	0.005326727
2310035C23Rik;Kiaa1468	0.004341889
5730469M10Rik;Fam213a	0.08303692
9030617O03Rik	0.05496452
AA467197;Nmes1	0.034494704
Abcb1a	0.083590193
Abcc2	0.052553808
Abcd3	0.09674549
Abcf2	0.018924959
Abr	0.00840842
Acaa1b	0.033770872
Acaa2	0.068441397
Acad8	0.048674576
Acadsb	0.01959085
Acadvl	0.004316284
Acat2;Acat3	0.077348071
Ace	0.04392316
Acox1	0.096103606
Acp6	0.078693495
Acsf2	0.001804103
Acsl5	0.041410815
Acss1	0.048476197
Actb	0.016441349
Actg1	0.065453002
Actl6a	0.011079111
Adprhl2	0.014992962
Adrm1;Gm9774	0.047075201
Adsl	0.025546046
Aer61	0.039703643
Agpat2	0.004874534
Ahcyl1	0.094231969
Ahnak	0.099001037
Ahsa1	0.002041363
Ahsg	0.037135514
AI314976	0.069663372
AI747448	0.033165405
Ak1	0.017615432
Akap12	0.061249262

Akap9	0.045110096
Alb	0.05743972
Aldh16a1	0.099986287
Aldh1a2	0.026329946
Aldh9a1	0.067142868
Alkbh5	0.015683712
Alyref	0.007995745
Amdhd2	0.042423818
Anapc1	0.040391894
Ank3	0.060750298
Ankrd17	0.038674297
Ankrd28	0.036924379
Ankrd44	0.001269242
Ano10	0.003091004
Ano6	0.039920113
Anp32a	0.020688191
Anp32e	0.0786442
Anxa2	0.026532914
Ap2b1	0.075529506
Ap3d1	0.0575507
Apex1	0.039386907
Apoa1	0.064048689
Apoa4	0.064847932
Apoe	0.071703605
Apoh	0.081876284
Apool	0.011186468
App	0.05226314
Arfgap3	0.078165034
Arfip1	0.086883055
Arglu1	0.003677077
Arhgap18	0.069241301
Arhgap5	0.024892305
Arhgef5	0.089016413
Arhgef7	0.096710786
Arid1a	0.07016305
Arl1	0.044365338
Armc8	0.076559215
Arpc4	0.040752594
Arpc5l	0.06699454
Arrb2	0.029185133
Asah1	0.044783304
Asah2	0.099141577
Ash2l	0.01515896

Aspn	0.008166235
Atg7	0.03632554
Atic	0.076077804
Atp1a1	0.064327526
Atp5j	0.038128279
Atp6v0d1	0.064950809
Atp6v1b2	0.012213611
Atp8a1	0.069346427
Atpif1	0.043949591
Atrx	0.025984582
B2m	0.000842869
Bag6	0.011258486
Baiap2l1	0.056091285
Basp1	0.024608462
Baz1b	0.008863213
Bcap31	0.006079891
Bcar3	0.096541834
Bcas1	0.077368809
Bcas2	0.001569767
Bdh1	0.082531755
Bpgm	0.091092804
Brix1	0.006856729
Bst2	0.012896171
Btaf1	0.031722855
Btf3	0.004377788
Bub3	0.018080773
Bud31	0.036629656
Bzw1	0.023360624
Bzw2	0.064937941
Ca4;Car4	0.009571377
Cacybp	0.005158438
Cald1	0.032536739
Calu	0.07234415
Calu	0.08306037
Camk1d	0.055106509
Camk2g	0.074318966
Canx	0.034407962
Caprin1	0.003731543
Carm1	0.01375688
Cars	0.051287214
Casp7	0.017534165
Casp8	0.085797908
Ccar1	0.002175601

Ccdc25	0.027934668
Ccdc58	0.02568065
Ccdc91	0.092274653
Cda	0.005842546
Cdc16	0.096553745
Cdc37	0.047094319
Cdc73	0.010878912
Cdk11b	0.004316844
Cdk5rap3	0.068849344
Celf1	0.019295449
Ces2a	0.036088747
Ces2c;Ces2d-ps	0.086130348
Ces2e	0.029190596
Cfl2	0.052468504
Chd4	0.011751057
Chga	0.066265721
Chgb	0.023989276
Chmp3	0.091301877
Cisd3	0.050245645
Clasp1	0.02657251
Clca1	0.087059231
Clca4	0.066069639
Clic6	0.045991837
Clip2	0.022625582
Clns1a	0.062977863
Clta	0.0984754
Cltc	0.08328334
Cmbl	0.060631674
Cmpk1	0.039609273
Cnn1	0.006410353
Cnot1	0.009886291
Col4a3bp	0.023686921
Cops5	0.008604785
Cops6	0.083223983
Coq6	0.004225186
Cox7a1	0.005440825
Cp	0.043596085
Cpsf1	0.03676513
Cpsf7	0.008214671
Crat	0.092193284
Crip1	0.077828227
Csde1	0.039638272
Csnk2a2	0.061519375

Csnk2b	0.00091558
Cstf3	0.012319341
Ctnnb1	0.074497127
Cttnbp2nl	0.004002103
Cul4a	0.071071988
Cul4b	0.017481452
Cwc22;OTTMUSG00000013393;Gm13695	0.034145382
Cwf19l1	0.007104642
Cyb5a;Cyb5	0.023994921
Cyp2b10	0.07494237
Cyp2c65	0.018541746
Cyp2d26	0.062911422
Cyp2j6	0.049950255
Cyp3a11	0.028532652
D10Wsu52e	0.019696631
D15Ertd621e;Fam91a1	0.093113695
Dak	0.083082571
Dars2	0.075292782
Dazap1	0.051397122
Dbnl	0.092715892
Dcaf8	0.055919764
Dcps	0.031744895
Dctn2	0.004175107
Dcxr	0.030421446
Ddb1	0.090965023
Ddi2	0.040674274
Ddx17	0.078245571
Ddx39b;Bat1a	0.01046677
Ddx5	0.07278925
Ddx6	0.036579318
Defa-rs1;Gm14851	0.00546185
Dgat1	0.0920102
Dgka	0.004389096
Dhrs1	0.03551789
Dhx15	0.011821233
Dhx9	0.009371435
Diap2;Diaph2	0.002136583
Dis3l2	0.04203323
Dkc1	0.06880433
Dlg3	0.043403505
Dnaja1	0.019832801
Dnaja2	0.069571588
Dnajb12	0.089724929

Dnajc13	0.098013999
Dnajc19	0.044680183
Dnajc2	0.001256778
Dnajc7	0.074329043
Dnajc8	0.042625059
Dnm1	0.017484116
Dnmt1	0.008716757
Dock10	0.065443528
Dock11	0.038070812
Dock2	0.068001509
Dopey2	0.009416701
Dpp9	0.066563155
Dpy30	0.097581335
Dstn	0.0252241
Dync1i2	0.08150174
Dynll2	0.041789712
Dynlt1c;Dynlt1;Dynlt1f;Dynlt1b	0.078087879
Ecsit	0.099128961
Eea1	0.020308127
Eed	0.013488746
Eef1a1	0.05692991
Eef1d	0.05837977
Eef1g	0.065807542
Eftud2	0.012114329
Ehd2	0.065516915
Ehhadh	0.093148363
Eif2a	0.051152443
Eif2ak2	0.001152547
Eif2s1	0.012066677
Eif2s3x;Eif2s3y	0.021986107
Eif3b	0.045167667
Eif3d	0.051200687
Eif3f	0.039731453
Eif3h	0.027222773
Eif3i	0.068178457
Eif3k	0.011705041
Eif3l	0.013164385
Eif4a3;Gm5576	0.00064669
Eif4e	0.02334529
Eif5a;Eif5a2	0.077584039
Elac2	0.074479144
Elmo1	0.089300375
Eno1	0.043073122

Enpp3	0.072753388
Ep300	0.042971117
Epb4.1l3;Epb41l3	0.050398784
Ephx2	0.019495831
Eppk1	0.040188484
Erap1	0.00899954
Ergic2	0.077750705
Erh	0.008676842
Esrp1	0.007152274
Ewsr1	0.022188199
Exosc1	0.002951161
Exosc2	0.004579824
Exosc3	0.035650964
Fahd1	0.038201432
Fam129a	0.098904939
Fam175b	0.086992796
Fam213b	0.010626351
Fam3d	0.063489891
Fam83h	0.072722835
Fam96b	0.033186869
Fam98b	0.080745292
Fam98c	0.078331407
Far2	0.099839533
Fars2	0.073328297
Fat1	0.075082875
Fermt2	0.049361736
Fermt3	0.013499391
Fhl2	0.010182286
Fkbp4	0.059845174
Fkbp5	0.077283572
Fmo4	0.064988482
Ftl1	0.076494943
Ftsjd2	0.020835604
G3bp2	0.045138686
Gak	0.081270437
Galm	0.08983563
Galnt1	0.057186872
Galnt2	0.089347574
Gap43	0.03321888
Gar1	0.08520208
Gars	0.068579943
Gbf1	0.093124634
Gbp1	0.00717997

Gbp2	0.01585753
Gbp4	0.000572046
Gbp6;Gbp10	0.000999595
Gbp7	0.000116657
Gbp9	0.009363462
Gc	0.04741171
Gclm	0.053491705
Gcnt3	0.04200427
Gemin4	0.091048382
Gemin5	0.022841889
Gimap4	0.010747817
Gk;Gyk	0.000324634
Gls	0.065059882
Glyr1	0.013867637
Gm10094;Sap18	0.023490902
Gm11062	0.052647869
Gm12250	0.004820947
Gm20425	0.049673613
Gm3168;gag-pro-pol;gag	0.023901226
Gm5428;Rpl6	0.068336174
Gm6104;Hmgb3	0.005459334
Gm8420	0.042512487
Gnb2l1	0.006152422
Gns	0.026316429
Gpd1	0.007636217
Gpd1l	0.091072271
Gphn	0.00133588
Gpr128	0.012988141
Gpx2	0.077663518
Grb2	0.071710294
Grpel1	0.099958933
Gsk3b	0.031108226
Gstk1	0.050941281
Gstm3	0.055314294
Gstp1	0.060664522
Gtpbp4	0.023112236
Guca2b	0.037827568
Guk1	0.020381799
Gusb	0.079909739
Gvin1	0.029623307
Gzma	0.047527836
Gzmb	0.014815745
H2-Aa	0.001559493

H2-D1	7.63E-05
H2-K1	3.95E-05
H2-T3;MumuTL;H2-T18	0.01873693
Hadha	0.045779789
Hadhb	0.020711946
Hba-a1;Hba-a2;Hba	0.069823655
Hbb-b1;Hbbt1	0.055062424
Hcfc1	0.026215741
Hectd1	0.091283014
Hip1r	0.001813078
Hkdc1	0.050601065
Hmbs	0.075162481
Hmgb1	0.039657455
Hmgb2	0.021663019
Hmgn5	0.042740102
Hnrnpa0	0.03294924
Hnrnpa1	0.007424298
Hnrnpa2b1	0.001375682
Hnrnpa3;Gm6793;Gm8991	0.006622381
Hnrnpab	0.044889763
Hnrnpc	0.002633899
Hnrnpd	0.0509909
Hnrnpf	0.016975639
Hnrnph1	0.021894251
Hnrnpl	0.028234131
Hnrnpul2	0.033978757
Homer1	0.058528603
Hpgd	0.025176184
Hsd17b12	0.007641057
Hsd17b2	0.010880195
Hsp90aa1	0.031204797
Hspa2	0.083265756
Hspa4	0.084880725
Hspa8	0.03138253
Hspa9	0.045287255
Hspb1;Hsp25	0.034712354
Hspe1;Hspe1-rs1	0.036240912
Hsph1	0.0871655
Htatip2	0.08710514
Iars	0.080247618
Ict1	0.060306307
Ide	0.040052554
Ido1	0.001083641

Ifi205b;Mnda	0.014792647
Ifi27l2b	0.046528966
Ifi47	0.000899135
Ifit1	0.034096695
Iigp1	0.051599776
Ikbkap	0.049549265
Ilf2	0.032700172
Ilf3	0.009133392
Ilk	0.077742992
Ilvbl	0.059629112
Inpp4a	0.062869863
Ipo4	0.000243047
Ipo5	0.062353328
Iqgap2	0.005604684
Irf8	0.093029072
Irgm1	0.01870015
Irgm2	0.01604477
Isg15	0.021372345
Ist1;2400003C14Rik	0.094967605
Isyna1	0.003384669
Itga6	0.050174138
Jakmip1	0.045658218
Kars	0.012327346
Kdm1a	0.00728476
Keap1	0.086492098
Khdrbs1	0.003914911
Kiaa0564	0.026328077
Kiaa0664	0.000655498
Kiaa1598	0.062651422
Kif13a	0.032284564
Kif5b	0.021881112
Kng1	0.07646019
Kpna3	0.027852454
Kpnb1	0.013153442
Krr1	0.025151554
Lamp2	0.093390577
Lancl2	0.094599109
Larp7	0.04992623
Lbr	0.006841386
Lcp1	0.073347928
Letmd1	0.005517469
Lgals3	0.014553612
Lgals3bp	0.002560582

Lgals9	0.018788953
Lias	0.027310893
Lima1	0.060635296
Lims2	0.002145401
Lman2	0.052772392
Lmnb1	0.028475758
Lmnb2	0.094625357
Lpgat1	0.077954272
Lpp	0.022280344
Lrpprc	0.010243724
Lrrc8c	0.023660828
Lrrfip2	0.064496727
Lsm4	0.011052159
Lsm7	0.028056267
Ly75	0.027864737
Magoh;Magoh-rs1;Magohb	0.01941154
Maob	0.049930118
Mapk1	0.014344896
Mat2a	0.018192467
Mat2b	0.087434597
Mbnl2	0.092291061
Mcat	0.001869614
Mccc1	0.009777397
Mcee	0.071561798
Mcm3	0.00343281
Mcm5	0.000154946
Mcu	0.092666021
Me1	0.096781941
Metap1	0.041588275
Metap2	0.053565285
Mettl7b	0.06919312
Mkln1	0.067544076
Mlkl	0.003689974
Mocs1	0.052475002
Mov10	0.024892951
Mpp7	0.091791184
Mrpl13	0.02842746
Mrpl15	0.066556067
Mrpl2	0.061227822
Mrpl20	0.084775682
Mrpl24	0.009988891
Mrpl37	0.054883774
Mrpl39	0.053316366

Mrpl40	0.041380927
Mrpl41;Gm6434	0.020408582
Mrpl45	0.027354369
Mrpl47	0.052589243
Msh6	0.073581493
Mta2	0.023363576
Mta3	0.045802979
Mtor	0.09986601
Mtus1	0.006905011
Muc13	0.062452214
Mug1;Mug2	0.052786416
Mybbp1a	0.072058577
Myg1	0.074477912
Naa15	0.000136479
Naa25	0.055008845
Naca	0.060777365
Nap1l1	0.067840377
Napg	0.068238141
Nars	0.030566054
Nasp	0.019169765
Nbas	0.034071358
Ncapd2	0.058352535
Ncbp1	0.001916102
Ncl	0.000758315
Ndufa12	0.089768781
Ndufa13	0.003395466
Ndufs6	0.027905695
Nfs1	0.068114821
Nfu1	0.04116977
Nmt1	0.032806097
Noc3l	0.04591156
Nomo1	0.065122568
Nop2	0.078797741
Npepps	0.088895723
Npl	0.000307244
Npm1;Gm5611	0.004879257
Nrd1	0.023329423
Nsfl1c	0.089848442
Nucb1	0.091062205
Nucb2	0.01371091
Nudc	0.092040957
Nudcd1	0.020224324
Nudt21	0.004146451

Nudt7	0.086483716
Numa1	0.02368074
Nup107	0.045146249
Nup210	0.073084585
Nup214	0.034254181
Nup37	0.033236595
Nup50	0.045078087
Nup85	0.021993539
Nup98	0.025241216
Nupl1	0.045439125
Oat	0.091796523
Ociad2	0.056090742
Optn	0.051100868
Pa2g4	0.045277443
Pabpc1	0.032551304
Paf1	0.008125706
Pafah1b3	0.073068911
Paics	0.09042864
Pak1ip1	0.006406701
Pak2	0.038431649
Parn	0.000525237
Parp14	0.027677972
Parp3	0.094183184
Parp9	0.019216389
Pcna	0.003265464
Pcnp	0.072980755
Pdcd11	0.073816877
Pdcd2	0.01281118
Pde5a	0.080377123
Pdia3	0.097520873
Pdk2	0.03195247
Pdk3	0.089413071
Pdlim3	0.065920023
Pdxdc1	0.058288287
Pecr	0.047052467
Pfdn2	0.042837895
Pfdn5	0.073679318
Pfdn6;H2-Ke2	0.027427225
Pfkm	0.017187827
Pgm5	0.048107759
Phyh	0.009868626
Pik3c2a	0.087857292
Pik3r1	0.099209331

Pklr	0.023969937
Pla2g4c	0.01539552
Plaa	0.039575952
Plbd1	0.085702865
Plcb3	0.049247125
Plcl2	0.095500962
Plin4	0.05037259
Plvap	0.089889143
Pmpca	0.070973949
Pnn	0.081085326
Pnp	0.069708789
Pnp2	0.060340887
Pnpt1	0.098112219
Ppat	0.009527598
Ppfia1	0.046655399
Ppia;Gm5160	0.082743704
Ppid	0.007878637
Ppie	0.0162198
Ppif	0.067973141
Ppih	0.025042581
Ppil1	0.031824934
Ppm1g	0.079537496
Ppm1h	0.0665295
Ppme1	0.063306731
Ppp1r10	0.01406842
Ppp1r12a	0.092355744
Ppp1r12b	0.037193521
Ppp1r9b	0.039196808
Ppp2r1b	0.011448771
Ppp2r2a	0.067482445
Ppp2r5a	0.041233541
Ppp3ca	0.014950488
Ppt1	0.06416228
Prkab1	0.0205115
Prkca	0.054668907
Prmt1	0.018684728
Prmt5	0.013512015
Prpf19	0.013694134
Prpf31	0.065444528
Prpf38a	0.004935781
Prpf38b	0.006141231
Prpf39	0.05934249
Prpf40a	0.004877425

Prpf8	0.004103806
Prpsap2	0.052322499
Prrc1	0.031349382
Psma1	0.019721793
Psma2	0.035747626
Psma3	0.037448391
Psma4	0.028190437
Psma5;Gm8394	0.004977707
Psma6	0.0315015
Psma7	0.028878987
Psmb1	0.010237802
Psmb2	0.007649879
Psmb3;Gm5356	0.033589671
Psmb5	0.05164374
Psmc3	0.006429835
Psmd1	0.045042974
Psmd10	0.020839532
Psmd11	0.017307906
Psmd12	0.085092047
Psmd13	0.081369143
Psmd2	0.007659516
Psmd6	0.035929149
Psmd7	0.011767368
Psme2	0.004447314
Psme3	0.0071004
Pspc1	0.003240408
Ptges3	0.042094501
Ptk2b	0.013685031
Ptprc	0.053468816
Ptprj	0.090916855
Ptrf	0.097463953
Ptrh2	0.072334204
Puf60	0.069870128
Pwp1	0.024660854
Pycard	0.014172394
Pzp;A2m	0.077954551
Q6PGB6-2, Q6PGB6-5	0.023967653
Rab10	0.025210836
Rab27a	0.087223497
Rab27b	0.01517187
Rab3a	0.056560711
Rab6a	0.073120235
Rabgap1l	0.079703066

Rabl3	0.027022671
Rad23b	0.075377251
Rad50	0.00567597
Rae1	0.025748502
Raly;C130057N11Rik	0.022963746
Rangap1	0.005332067
Rap2b	0.06537738
Rb1cc1	0.027563372
Rbbp4	0.025396491
Rbbp7	0.023336843
Rbm39	0.017894398
Rbm47	0.018065807
Rbm8a	0.001507795
Rbmx	0.002528588
Rbp2	0.048145515
Rbx1	0.073324829
Rcc1	0.000314123
Rcc2	0.004646407
Rdh11	0.027508581
Rdh7	0.086388355
Rdh9;Rdh16	0.040269067
Rdh9;Rdh19;Rdh1	0.032527202
Rfc3	0.092683728
Rfk	0.020373387
Rg9mtd1	0.005612565
Rhoc	0.054707187
Rnaset2	0.033330119
Rnf213	0.009970486
Rpa1	0.008892388
Rpap3	0.028710401
Rpia	0.006753104
Rpl10a	0.061927648
Rpl12	0.013544571
Rpl13a;Rpl13a-ps1	0.071510094
Rpl19	0.011206562
Rpl22l1	0.007575828
Rpl32	0.062927451
Rpl4	0.002289311
Rpl5	0.01639681
Rpl9-ps6;Rpl9	0.090212726
Rpn1	0.085979544
Rpn2	0.070232894
Rps15a	0.044848591

Rps19	0.034137281
Rps2;Rps2-ps5;Rps2-ps6;Gm6576	0.032739039
Rps21	0.0281132
Rps24	0.096046631
Rps25	0.008236064
Rps3	0.055465044
Rps9	0.008311544
Rpsa;Rpsa-ps10	0.036468125
Rragc	0.030035684
Rras	0.074842048
Rrbp1	0.033879448
Rrm1	0.02177395
Rrp12	0.023393066
Rsu1	0.012634405
Ruvbl1	0.023012643
Ruvbl2	0.001566269
Rxra;Rxrg	0.013749942
S100a10	0.02913272
S100a13	0.081132563
Sacm1l	0.063485061
Safb	0.028883206
Samhd1	0.080199291
Sart3	0.064951036
Scarb2	0.040884173
Scin	0.033081736
Scly	0.01551271
Scp2	0.015007581
Sdpr	0.040041921
Sec16b	0.083714861
Sec31a	0.064695617
Sec61a1;Sec61a2	0.080100966
Sectm1b	0.078470284
Seh1l	0.03315999
Sel1l	0.074107647
Selrc1	0.094406047
Sep11	0.083629136
Serhl	0.011413826
Serpina1a;Serpina1c	0.067108622
Serpina3k	0.043330724
Serpina3m	0.046637285
Serpinb6b	0.076699676
Serpinb9	0.044272423
Serpinh1	0.080853122

Sf3b14	0.07577021
Sf3b3	0.009396086
Sf3b4	0.057987775
Sgta	0.085446114
Shmt2	0.030994098
Shoc2	0.067699923
Skiv2l2	0.001674201
Slc12a6	0.028036027
Slc16a1	0.006003945
Slc25a10	0.089687777
Slc25a22	0.092383811
Slc25a23	0.059887694
Slc25a24	0.031185217
Slc25a3	0.065712503
Slc25a5	0.029636223
Slc27a2	0.065232674
Slc2a2	0.03640538
Slfn9	0.087963961
Sltm	0.068015593
Smad4	0.008760835
Smarca4	0.010301062
Smarca5	0.058948018
Smarcc1	0.000706862
Smarcc2	0.058002099
Smarce1	0.010952332
Smc1a	0.03128206
Smc2	0.014507712
Smc3	0.001358431
Smc4	0.004486617
Smek1	0.020486566
Smpdl3b	0.05372749
Sncg	0.062793914
Snf8	0.061554997
Snrnp200	0.000396933
Snrnp40	6.31E-05
Snrnp70	0.008650835
Snrpd1	0.018308301
Snrpd2;Gm5449	0.016872036
Snrpd3	0.013068505
Snx27	0.012567288
Snx5	0.016113995
Snx6	0.030854865
Sorbs2	0.074758589

Sord	0.06332095
Sp100	0.030201539
Sptlc1	0.024827275
Sptlc2	0.010577996
Sqrdl	0.028403349
Srp68	0.096705605
Srsf1	0.002428032
Ssb	0.019860209
Ssbp1	0.036838224
Stat1	0.000549893
Stat3	0.001265989
Stat5b	0.054457338
Stim1	0.066944903
Stip1	0.001050048
Stk39	0.084023022
Strap	0.049074044
Strbp	0.040882567
Stt3a	0.003953766
Stt3b	0.057736275
Stx12	0.030878352
Stx4	0.035762577
Sub1	0.013528548
Sun1	0.094325532
Supt5h	0.015399967
Supt6h	0.019132443
Syncrip	0.008672996
Tapbp	0.000325812
Tax1bp1	0.016366225
Tbc1d8b	0.052799175
Tbcd	0.095696432
Tbl1x	0.045998159
Tcerg1	0.029139913
Tes	0.051992279
Tfrc	0.008626085
Tgtp2;Tgtp;Tgtp1	0.039240849
Thy1	0.006129291
Thyn1	0.004401958
Tial1	0.028642205
Timm10	0.015710858
Timm13	0.033621988
Timm50	0.030878029
Timm9	0.087395413
Tjp2	0.036448689

Tmbim6	0.011115375
Tmod3	0.078984737
Tmtc3	0.034548151
Tnpo2	0.021733867
Tns1	0.04020382
Tom1l1	0.072567125
Top2a	0.012968547
Tpd52	0.047207201
Tpd52l2	0.037876107
Tpm3-rs7;Tpm3	0.013371858
Tpr	0.001123431
Trappc11	0.016192476
Trim12c;Trim5	0.060722845
Trip11	0.073312453
Trmt1l	0.025507248
Trpm4	0.083851077
Tsfm	0.036874018
Tsnax	0.037864976
Ttc35	0.08266006
Ttr	0.025823193
Tuba4a	0.089875572
Twf1	0.096111398
Twf2	0.064797891
Txn	0.049181886
Txndc9	0.016710002
Txnl1	0.066904771
U2surp	0.00266084
Uaca	0.044231888
Uba7	0.004963192
Ubqln1	0.077691966
Ubr7	0.036220149
Ubtf	0.004357029
Ubxn1	0.098754071
Uchl3;Uchl4	0.089185109
Uchl5	0.009077822
Ugt1a7c;Ugt1a8	0.079920002
Ugt2b34	0.09524211
Umps	0.021891411
UPF0444 transmembrane protein C12orf23 homolog	0.032502251
UPF0668 protein C10orf76 homolog	0.077738356
Upf1	0.085168755
Upp1	0.001237977
Uso1	0.0044575

Usp10	0.099159258
Usp14	0.05178295
Usp19	0.020589454
Usp24	0.045848632
Usp39	0.002739509
Usp7	0.015998971
Usp9x	0.09445796
Utp20	0.033350033
Vars	0.023310377
Vprbp	0.002393655
Vps29	0.086966612
Vwa5a	0.051429042
Wars	0.008221048
Wdr11	0.032958478
Wdr12	0.002923313
Wdr3	0.013904769
Wdr33	0.047075738
Wdr82	0.018312756
Xdh	0.023627799
Xpo1	0.004743864
Xpo4	0.04501831
Xpot	0.031489278
Xrn1	0.089141261
Xrn2	0.053214064
Yars	0.056937485
Yars2	0.054302342
Yes1	0.047162182
Ythdf2	0.083149079
Ythdf3	0.069400987
Ywhae	0.08118583
Ywhah	0.032118654
Zfp207	0.037987394
Zfr	0.037763741
Znf326;Zfp326	0.008370443

Supplemental Table 10. Apobec-1 C-to-U RNA editing targets with altered protein expression

C-to-U RNA editing targets	RNA-seq	WT/*Apobec-1*$^{-/-}$ Fold protein expression	WT/*Apobec-1*$^{-/-}$ Fold RNA expression (FPKM)
1. Cyp3a11	16/16%	6.6	3.8
2. Cyp2c65	**50%**	4.2	2.4
3. Rfk	16%	3.2	1.3
4. Tes	25%	3	1.5
5. Abcd3	15/21%	3	1.4
6. Hpgd	36%*	2.3	1.1
7. Cyp4v3	**49%**	2.2	1.6
8. Pde5a	11%	2.1	1.2
9. Yme1l1	**21%**	2.1	1.3
10. Cyp2j6	**23/24%**	2	2.4
11. Bdh1	15%	1.9	1
12. Lpgat1	12%	1.8	1.9
13. Dpyd	13/**13**/**38**/40*/13%	1.8	1.3
14. Ehhadh	10%	1.6	1.3
15. Iqgap2	32/12/30*/29%	1.6	1.1
16. Letmd1	16%	1.6	1.13
17. Gak	20%	1.5	1.4
18. Ank3	13%	1.4	1.9
19. Slc25a24	12/14/12%	1.3	1.1
20. Tbc1d8b	12/25%	1.3	1.2
21. Acadsb	17%	1.3	0.9
22. Kif5b	11%	1.3	0.9
23. App	**34**/16/13/14/**30**/11/14/22%	1.2	1.5
24. Vwa5a	14%	0.8	1
25. B2m	22/13/28%	0.5	0.9
26. Ido1	**18%**	0.2	0.7

Bold numbers indicates editing sites that were confirmed by Sanger sequencing. Asterisks indicate editing sites that were not validated by Sanger sequencing. RNA targets indicated in blue show higher protein expression in wild-type mice than in *Apobec-1*$^{-/-}$ mice. RNA target indicated in red show reduced protein expression in wild-type mice. Red lines mark the 2-fold change in protein abundance cut-off.

Supplemental Table 11. miRNA seed sequence in Apobec-1 C-to-U RNA editing targets with altered RNA and protein expression

Gene	Editing site	*Apobec-1* $^{-/-}$ miRNA seed match (C)	WT miRNA seed match (U)
1. Cyp3a1	Chr5: 146666370	mmu-miR-185-5p<br>mmu-miR-1935<br>mmu-miR-298-5p	mmu-miR-15a-5p<br>mmu-miR-15b-5p<br>mmu-miR-16-5p<br>mmu-miR-195a-5p<br>mmu-miR-6343<br>mmu-miR-411-5p
	Chr5:146666372	mmu-miR-3092-5p<br>mmu-miR-706	mmu-miR-186-5p<br>mmu-miR-330-3p<br>mmu-miR-466-3p<br>mmu-miR-6403<br>mmu-miR-6416-3p
2. Cyp2c65	Chr19:39168358	mmu-miR-15b-5p<br>mmu-miR-196b-5p<br>mmu-miR-199a-3p<br>mmu-miR-199b-3p<br>mmu-miR-3068-5p<br>mmu-miR-325-5p<br>mmu-miR-5117-5p	mmu-miR-3472
3. Rfk	Chr 19:17475766	mmu-miR-150-3p<br>mmu-miR-3101-5p<br>mmu-miR-33-5p<br>mmu-miR-34b-5p<br>mmu-miR-5129-5p<br>mmu-miR-547-3p	mmu-miR-144-5p<br>mmu-miR-200b-3p<br>mmu-miR-200c-3p<br>mmu-miR-3074-1-3p
4. Tes	Chr6:17055467	mmu-miR-3105-5p<br>mmu-miR-335-5p<br>mmu-miR-450a-1-3p	mmu-miR-142-5p<br>mmu-miR-320-3p<br>mmu-miR-330-3p<br>mmu-miR-340-5p<br>mmu-miR-5619-3p<br>mmu-miR-6240
5. Abcd3	Chr3:121462929		mmu-miR-190a-3p<br>mmu-miR-200b-3p<br>mmu-miR-200c-3p<br>mmu-miR-369-3p<br>mmu-miR-374b-5p<br>mmu-miR-429-3p<br>mmu-miR-466a-3p<br>mmu-miR-466b-3p

	Chr3:121462991		mmu-miR-466c-3p<br>mmu-miR-466e-3p<br>mmu-miR-466l-3p<br>mmu-miR-466p-3p<br>mmu-miR-467a-3p<br>mmu-miR-669b-3p<br>mmu-miR-669f-3p<br>mmu-miR-669m-3p<br>mmu-miR-302b-5p<br>mmu-miR-302d-5p
6. Hpgd	Chr8:58799127	mmu-miR-3112-5p	mmu-miR-421-3p
7. Pde5a	Chr3:122560887	mmu-miR-100-3p<br>mmu-miR-21a-5p<br>mmu-miR-21c<br>mmu-miR-27b-5p<br>mmu-miR-320-3p<br>mmu-miR-708-5p	mmu-miR-3066-3p<br>mmu-miR-6374
8. Cyp2j6	Chr4:96183317		mmu-let-7a-1-3p<br>mmu-let-7b-3p<br>mmu-let-7c-2-3p<br>mmu-let-7f-1-3p<br>mmu-miR-1957b<br>mmu-miR-1a-3p<br>mmu-miR-206-3p<br>mmu-miR-3084-5p<br>mmu-miR-344d-3p<br>mmu-miR-369-3p<br>mmu-miR-374b-5p<br>mmu-miR-410-3p<br>mmu-miR-539-3p<br>mmu-miR-6387<br>mmu-miR-669e-3p<br>mmu-miR-98-3p
	Chr4:96183404		mmu-miR-129-5p<br>mmu-miR-376a-5p
9. Ido	Chr8:25694791	mmu-miR-145a-5p<br>mmu-miR-145b<br>mmu-miR-19a-5p<br>mmu-miR-29b-1-5p<br>mmu-miR-382-5p<br>mmu-miR-669b-5p<br>mmu-miR-669c-5p	mmu-miR-1941-3p<br>mmu-miR-216a-5p<br>mmu-miR-29a-5p<br>mmu-miR-374b-5p<br>mmu-miR-872-5p

RNA indicated in blue are upregulated in editing-competent wild-type animals.
RNA indicated in red is downregulated in wild-type mice.

Supplemental Table 12. Primer sequences for PCR amplification of intestinal 3'UTR Apobec-1-dependent RNA targets

RNA	Chr	Position	Forward Primer	Reverse Primer
Abcb7	X	101478733 (-)	CTGTGTTTGAAGTCTGACATCCATTTG	GATAGCCAATGCAGTGCACTGAAGTTG
Actr2	11	19963383 (-)	GGTACCCAAGTGCGGGGTCTTCC	GACCCTCAAGGCATTGGTG
Aldh6a1	12	85772761 (-)	GGTCATCCTCTAGTTGAGCC	GGTGACTGGATACTGTCAAAGC
Ank3	10	69486962 (+)	GGAAACTCACATTCATTCCCGGCAC	GGACGCCTGTCCTTGGATGCATTTG
App	16	84954758 (-)	GGCGTCTAGCTGCTTCTCCTACG	GGTGTGAGTGACCAGATGAGGTG
App	16	84955113 (-)	GGCTTTTGACAGCTGTGC	GGAGACCAGCAGAACACTCCC
Atf2	2	73654730 (-)	GGTCAGAGCCTCCATGTGTCAA	GGACTCAAAATGCTAGG
Atp6ap2	X	12193607 (+)	GGCTACGTAGTGCAGCTG	GGTCCATTTGCACATGAACAG
Atp11c	X	57477477 (-)	GGACCTTGGTAAGATCCTC	GGTTAACTGAGGCATGACTC
Bche	3	73442586 (-)	GGGTTAGCACTTGAGG	GGCAAAGCCCCTCTCACC
BC003331	1	152208563 (-)	GGCAACTCAGTCACATGCATAG	GGAATGACCACATTTAAGCCTTAC
BC013529	1	152209582 (-)	AAATTCCCAAGACCTTTACAG	AAATGTTAAGTTCTCCAGCTT
B2m	2	121978638(+)	GCACGCAGAAAGAAATAGC	GGGCACAGTGACAGACTTCAATTAGGC
Casp6	3	1229616676(+)	GGTGGAAGACTTTCACCCATC	GGGGAGGCAAAGTAGTTGG
Ccny	18	9315769 (-)	GGAATGCCATGCTATGCCCTTGG	GGACATGGCTCCTCATGAGGTCAG
Cd36	5	17288955 (-)	GGCTACATCTTTGGTAAAGCC	GGCCTTGCTGTAGCCAAGAACTCC
Clic5	17	44416335 (+)	GGGCCCTTCTACAAATAAGCC	GGTTGATGCAGTGCTAGAGATCTGACC
Cmtm6	9	114658289(+)	CAGGCTTTCCGGTTAGGAAG	GTGATCAGATATAAGCAACGTAG
Cnih	14	47395982 (-)	GGTCCTGTTTCCAAGAGTAGCC	GGAGGGTTATGGCTGTTCC
Cyp2c65	19	39168358 (+)	TTAAGGCAATTATTAGGGTTACTGC	AAAGTTACCTTCAGCTGCATAGTGA
Cyp4v3	8	46391931 (-)	GGAGACATGAAGATGACCCC	GGTGACTGTACCTTGACAGG
Ddx60	8	64516163 (+)	GGTTGCCTCTAATGTACTTGGTG	GGCAGTGTACAGTGGATTAATAG
Dek	13	47181166 (-)	GGTAGAACTTGCTGCTGACTC	GGTCATCTGAACAGCTGTGCCCTA
Dpyd	3	119135667(+)	GGCTAGATTACACAGCGCTG	GGAAGCACTTGAGAATGATACC
Dpyd	3	119135669(+)	GGCTAGATTACACAGCGCTG	GGAAGCACTTGAGAATGATACC
Dpyd	3	119134696 (+)	CAACACCTTATCAACCAAAGAG	GTCACTGGCATTAGACATTG
Fgl2	5	20883372 (+)	AATATAACGTAGATTTTAAAGCC	TCAGGATGAACACATAGTATT
Fmn1	2	113556683(+)	ACCAAATGAAGTATTTATT	AGAATATCAATAGATTAGGAT
Gramd1c	16	43981376 (-)	GGACATCAATGCTCATAATGAACTAC	GGTGCTGACACAAGTGCCTGAGTG
Herc2	7	63486942 (+)	GGACCTAGTGTTTACTACAGTG	GGTGCACCTGACCCAGACCAG
Hprt1	X	50374459 (+)	CCTAGTAAAGCTTTTTGCATGA	CCATCTTTCTCTCCTGAATATATAA
Iqgap2	13	96397211 (-)	CTGATGAGTCCCACCAAGAG	GTTCTAAGCCCTGTCTTCTG
Iqgap2	13	96397289 (-)	CTGATGAGTCCCACCAAGAG	GTTCTAAGCCCTGTCTTCTG
Kctd12	14	103379573 (-)	GGACAGTCAGCCCTTACTTAGGTC	GGCTCACACTGAAGTACTTATTTGC
Lrba	3	86586529 (+)	GGCGACCAGGTACTGTGTAAGCAG	GACCTCTGCTCAAATATTTTCACAG
Lrrc19	4	94304303 (-)	GCAGTGTTCGGGAAACTACAGACTGC	GGGACCTGAACATGCTATATAGATGCCC
Man2a1	17	65104330 (+)	CATTGTAATTATCATCCCGT	GAAGACAATGCACAGGATAA
mCG_2776	6	86586529 (+)	AAGTAAAGCTGAGCAGGATTG	AAATGAGTATGCAGAGCACAT
Mcmbp	7	135841366 (-)	GGCCAGACTATGAAGTGCCAGC	GGGCAGTGTTCCCCTCAATCC
Mfsd7b	1	192830761 (-)	AAGCTTACATTCATGACATTT	GATACTAAGTTCTTTCAGTACATT
Mtmr2	9	13610423 (+)	CACCTGAGAGCACACGCATT	AATACACAAATGGAGTAGATGACAGC
Nr3c1	18	39571801 (-)	GGCAATAATGTGCATAGAGGTTC	GGAGCTGCTCTCAAACAGTAC
Nr1d2	14	19036726 (-)	CTAACAGTTAATAAATGACAT	AATGTAGATATGAAATCTATA
Ptpn3	4	57203753 (-)	GGAGCAACCACAGGATTGTGTGC	GCATAGCTTGAACGC
Rab1	11	20125336 (+)	GGCATGTTTAGATGTTGGG	GGTGAAGTCTCTACACAGC
Rb1	14	73595382 (-)	GGTCAAGGGCTTACCATACTTCTAGG	GGCTCTGAACAACTAG
Reps2	X	158851906 (-)	ATTTTACAATAGTTTAGACATTGG	GATATTCCCATGGGCATATT
Rnf128	X	136207009(+)	GGGGTTTGTCTCCAGGTGC	GGTAGTTTCTATCCTGTGC
Rrbp1	2	143811725 (-)	GGAAGCAACCCTGAAGAAGGC	GGGAATAAGGGATACAGC

Sep15	3	144259976(+)	GAGCGAGAAGTTGGAACGC	GGACTGTGGTGCTACTTCAGC
Serinc1	10	57235791 (-)	GGCGATGTTAACAATTCTGC	GGCTGGAACATGAAGATGAACTGC
Sh3bgrl	X	106355759(+)	GTTCTGAGTTCTTCCTTCAG	GATGAGAACCTTAAGCTACACAG
Siglec5	7	50614573 (+)	TAATGCATACTTGAATTTTACT	CTTTATATATTAATTTTATGCCC
Skil	3	31018375 (+)	ACTCATAATCTTCTTGTACAGTT	ACCTACCACATATTCTTAGCT
Slc4a4	5	89668527 (+)	CAACATGGTGGAACAAAAT	AAATGCATAAAGTTACTTTATTTTAA
Sult1d1	5	87984364 (-)	GGCCTCCTAGAGGAAG	GGTGTGATCCAAAACCC
Tmbim6	15	99239051 (+)	GGCCGTCAGCCTTTCCCAGC	GGTCTCCTTTCCTATCCTCC
Tmem30a	9	79617629 (-)	GGTGGGCAGCTGCTTGGCAGTC	GGCATCAACATCTCACATC
Tmem135	7	96290044 (-)	CCAATCAGAAATACAGAAGCTTT	ATCCTTTGCAAATCCTAAAGT
Tmem195	12	38308269 (+)	GGTCCTGTCAGTTCTCCGTCCTGC	GGATCAGCTATCAAACCAAGAAGAC
Ttc9c	19	8885447 (-)	GGCCCTGGGTTTAATTCCCCAGTGC	GGTTTTCCCACAGAGCTGTTATCTCCAGCCC
Usp25	16	77116537 (+)	GGGGTGGACATTGGATGATGC	CGGTGGTAACATGATTACC
Yes1	5	32989151 (+)	GGTCCTCTTGCCCTGATGTCTACTC	GTGTGTTAAAGTTCCACGTTAAG
Yme1l1	2	23052720(+)	GGCAGAAGCATTGCTGACC	GGCACAATTTTTACTCACTTGGAG
06010010O12Rik	18	36562329 (+)	CTCACCTGATCACCTGATGA	TAAAGATCAGTAAGAAATGAGTTT
1110020G09Rik	15	9038469 (+)	TTTAGAGGCAGGTTAAATAAAGGA	GTTCCCATGGTTTCAATCAATA
2010106E10Ri*k*	X	109671648(+)	TGCAAACATGATACCCAACTATTA	AGATAACAAATATCTGCATCTGGT

Supplemental Table 13. Primer sequences for PCR amplification of hepatic 3'UTR Apobec-1-dependent RNA targets

RNA	Chr	Position	Forward Primer	Reverse Primer
Abcc9	6	142538042 (-)	GGTACCTCTATAAAGCTAACCC	GGCTGGGAATACCTGGTACCTC
Abcc9	6	142538035 (-)	GGTACCTCTATAAAGCTAACCC	GGCTGGGAATACCTGGTACCTC
Cd36	5	17288955 (-)	GGCTACATCTTTGGTAAAGCC	GGCCTTGCTGTAGCCAAGAACTCC
Colec10	15	54297696 (+)	GGGCACCTACCCTCCCATGTGC	GCATCTGCTAGAATCACCG
Colec10	15	54295026 (+)	GGATGCCCTGCTTATGGGTTGTGAC	GGCCTTCAAAATACCCTATGCC
Cybb	X	9012717 (-)	GGCTGGATGAGGCAACCCAGG	GGGAGGGTTGTAACTGATTGAC
Cybb	X	9012852 (-)	GGCTGGATGAGGCAACCCAGG	GGGAGGGTTGTAACTGATTGAC
Cybb	X	9013390 (-)	GGTGGCTAACACTTCTAGTTCC	GGAGAAGGATTCTTGTAGCAAC
Dcn	10	96980667 (+)	GGAAACTACAAGTAACCCTCAGACGGC	GGCTTCACCAATAATAAAGG
Dcn	10	96980535 (+)	GGAAATTCAAGCACACTGTGC	GAACTTACATACTCAAATAAGGC
Mpeg1	10	12539179 (+)	GGATGGCTTCCATGGGTTATCTGG	GGCCTACTCTGTGCACAAGAGC
Ube2l3	16	17152203 (-)	GGAGCAGCCACCCACGCCTG	GGACAGAATGGGGTCACAGC

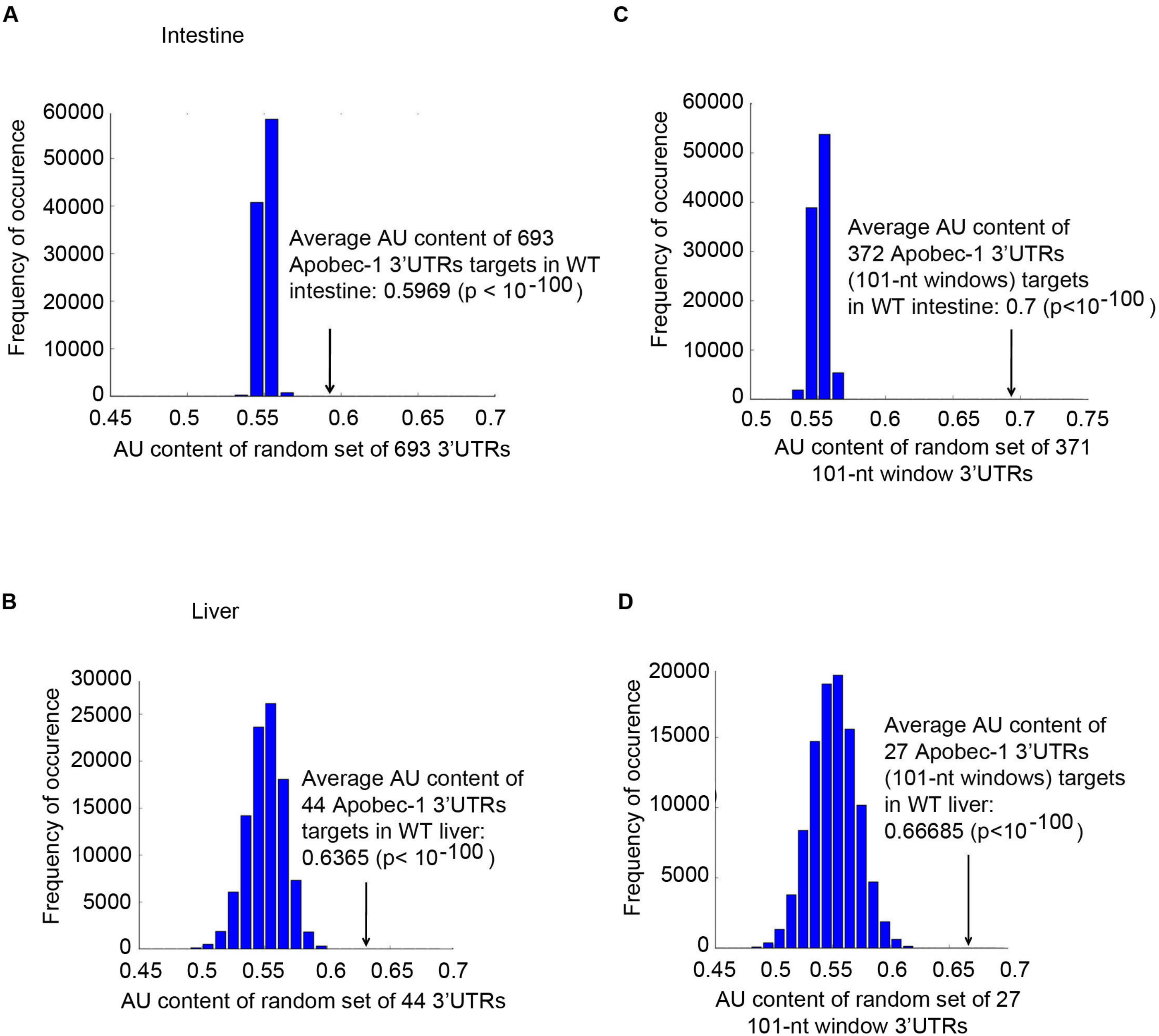

Supplemental Figure 1

E

WT intestine

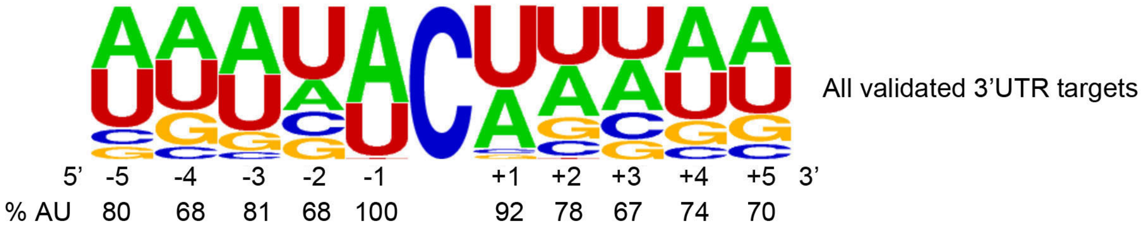

F

WT Liver

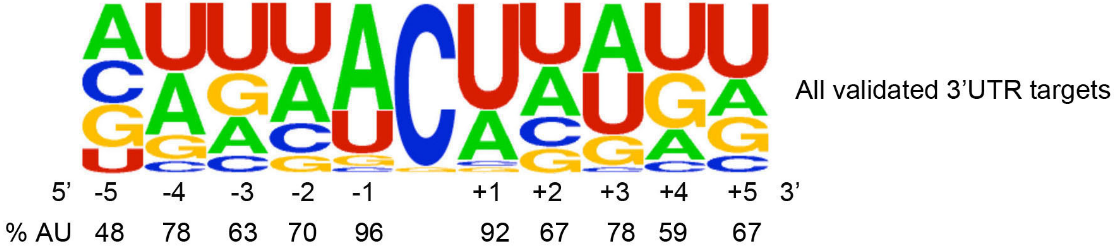

Supplemental Figure 1

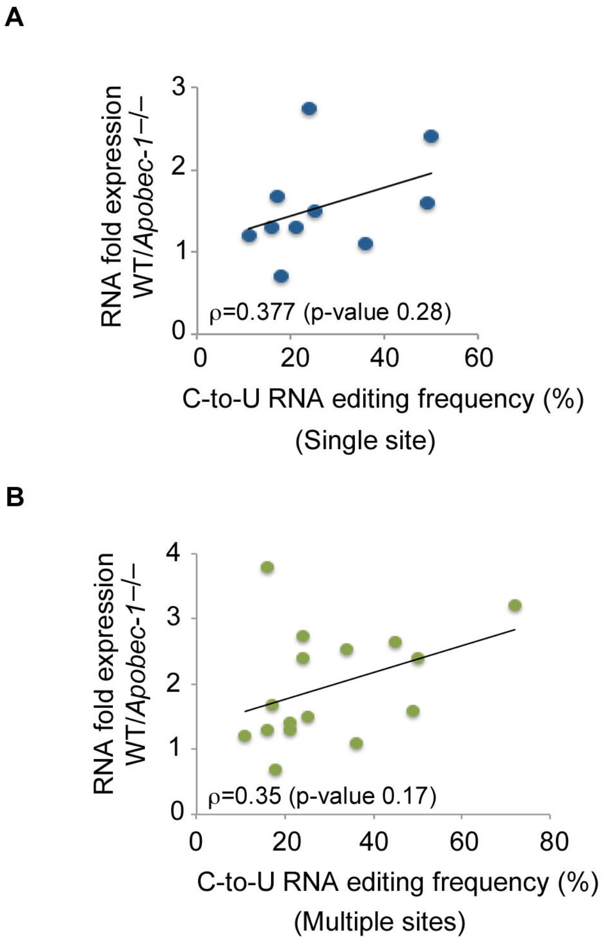

Supplemental Figure 2
